# Supplementary material for: School-Based Interventions to Support Healthy Indoor and Outdoor Environments for Children: A Systematic Review
Source: Int J Environ Res Public Health. 2023 Jan 18;20(3):1746. doi: 10.3390/ijerph20031746 (PMC9914556; doi:10.3390/ijerph20031746)
Supplement: Supplementary file 1 [file ijerph-20-01746-s001.zip › Supplementary Material S1 full search strategy for all databases.pdf]

Search strategy

(* indicates truncation at the end of the Word)

PubMed

Population

1. Child[Mesh:NoExp]

2. child*[Title/Abstract] OR schoolchild*[Title/Abstract] OR school aged child*[Title/Abstract] OR school child*[Title/Abstract]

3. 1 OR 2

Setting

4. Schools[Mesh:NoExp]

5. School Health Services[Mesh:NoExp]

6. school[Title/Abstract] OR schools[Title/Abstract] OR primary school*[Title/Abstract] OR school-based[Title/Abstract] OR elementary school*[Title/Abstract] OR middle school*[Title/Abstract] OR kindergarten[Title/Abstract] OR pre school*[Title/Abstract] OR preschool*[Title/Abstract] OR pre-school*[Title/Abstract]

7. 4 OR 5 OR 6

Intervention

8. environment[MeSH Terms:noexp]

9. Environment Design[Mesh]

10. Public Facilities[Mesh]

11. Walking [MeSH Terms:noexp]

12. Bicycling[MeSH Terms]

13. Air Pollution[MeSH Terms:noexp]

14. air pollution/adverse effects[MeSH Terms:noexp]

15. air pollution/prevention and control[MeSH Terms:noexp]

16. particulate matter/adverse effects[MeSH Terms:noexp]

17. Particulate Matter[MeSH Terms:noexp]

18.Air Pollutants[MeSH Terms:noexp]

19.air pollution, indoor[MeSH Terms:noexp]

20. Noise[MeSH Terms:noexp]

21. noise/adverse effects[MeSH Terms]

22. noise/prevention and control[MeSH Terms]

23. noise, transportation[MeSH Terms]

24. noise, transportation/adverse effects[MeSH Terms]

25. noise, transportation/prevention and control[MeSH Terms]

26. forests[MeSH Terms]

27. trees[MeSH Terms]

28. parks, recreational[MeSH Terms]

29. "Urban Renewal"[Mesh:NoExp] OR "City Planning"[Mesh:NoExp] OR "Built Environment"[Mesh:NoExp]

30. "active transport*"[Title/Abstract] OR "active travel*"[Title/Abstract] OR "safe route*"[Title/Abstract] OR "pavement*"[Title/Abstract] OR "sidewalk*"[Title/Abstract] OR "bicycle paths"[Title/Abstract] OR "biking"[Title/Abstract] OR "walking paths"[Title/Abstract] OR "pedestrian crossing*"[Title/Abstract] OR "traffic calming"[Title/Abstract] OR "traffic lights"[Title/Abstract] OR "pedestrian*"[Title/Abstract] OR "cycling"[Title/Abstract] OR "bike-riding"[Title/Abstract] OR commuting [Title/Abstract] OR commute [Title/Abstract] OR commuters [Title/Abstract]

31. (air pollut*[Title/Abstract] OR air quality[Title/Abstract] OR classroom ventilation[Title/Abstract] OR coarse particle*[Title/Abstract] OR Soot[Title/Abstract] OR black smoke[Title/Abstract] OR black carbon[Title/Abstract] OR elemental carbon[Title/Abstract] OR air clean*[Title/Abstract])

32. (road traffic nois*[Title/Abstract] OR traffic nois*[Title/Abstract] OR noise pollution[Title/Abstract] OR noise exposure[Title/Abstract] OR (transport*[Title/Abstract] AND Noise[Title/Abstract]))

33. "open space*"[Title/Abstract] OR "playing field*"[Title/Abstract] OR "green space*"[Title/Abstract] OR "greenspace*"[Title/Abstract] OR "greenness"[Title/Abstract] OR "greenery"[Title/Abstract] OR "recess"[Title/Abstract] OR "schoolyard*"[Title/Abstract] OR "school garden*"[Title/Abstract] OR "school ground*"[Title/Abstract] OR "outdoor teaching"[Title/Abstract] OR "outdoor learning"[Title/Abstract] OR (("nature"[Title/Abstract] AND ("acess"[Title/Abstract] OR "contact"[Title/Abstract] OR "recess"[Title/Abstract])) OR "green schoolyard*"[Title/Abstract] OR "nature-based"[Title/Abstract] OR "forest school" Title/Abstract] OR "forest schools" Title/Abstract])

34. 8/or33

Population, setting, and interventions

35. 3 AND 7 AND 34

Filters: AND ((humans[Filter]) AND (2010:3000/12/12[pdat]) AND (english[Filter]))

Search strategy PsycInfo/Green File

Population

1. TI ((child* OR schoolchild* OR "school aged child*" OR "school child*")) OR AB ((children OR child OR schoolchild* OR "school aged child*" OR "school child*")) OR MA ((child* OR schoolchild* OR "school aged child*" OR "school child*"))

Setting

2. TI ((school* OR "primary school*" OR school-based OR "elementary school*" OR "middle school*" OR kindergarten OR "pre school*" OR preschool* OR pre-school*)) OR AB ((school OR schools OR "primary school*" OR school-based OR "elementary school*" OR "middle school*" OR kindergarten OR "pre school*" OR preschool* OR pre-school*)) OR MA ((school* OR "primary school*" OR school-based OR "elementary school*" OR "middle school*" OR kindergarten OR "pre school*" OR preschool* OR pre-school*))

Intervention

3. TI (“built environment” OR "environment design" OR "public facilities" OR "Urban Renewal" OR "City Planning") OR AB (“built environment” OR "environment design" OR "public facilities" OR "Urban Renewal" OR "City Planning") OR MA (“built environment” OR "environment design" OR "public facilities" OR "Urban Renewal" OR "City Planning")

4. TI (("air pollut*" OR "air quality" OR "classroom ventilation" OR "coarse particle*" OR Soot OR "black smoke" OR "black carbon" OR "elemental carbon" OR "air clean*" OR "particulate matter" OR ("air pollution" N2 indoor) OR ("air pollution" N2 outdoor) OR ("air pollution" N3 prevention) OR ("air pollution" N3 control))) OR AB (("air pollut*" OR "air quality" OR "classroom ventilation" OR "coarse particle*" OR Soot OR "black smoke" OR "black carbon" OR "elemental carbon" OR "air clean*" OR "particulate matter" OR ("air pollution" N2 indoor) OR ("air pollution" N2 outdoor) OR ("air pollution" N3 prevention) OR ("air pollution" N3 control))) OR MA (("air pollut*" OR "air quality" OR "classroom ventilation" OR "coarse particle*" OR Soot OR "black smoke" OR "black carbon" OR "elemental carbon" OR "air clean*" OR "particulate matter" OR ("air pollution" N2 indoor) OR ("air pollution" N2 outdoor) OR ("air pollution" N3 prevention) OR ("air pollution" N3 control)))

5. TI ((noise OR "noise exposure" OR "road traffic nois*" OR "noise pollution)) OR ((noise OR "noise exposure" OR "road traffic nois*" OR "noise pollution))

6. TI (("active transport*" OR "active travel*" OR "safe route*" OR pavement* OR sidewalk* OR "bicycle paths" OR (biking N2 commut*) OR "walking paths" OR "pedestrian crossing*" OR "traffic calming" OR "traffic lights" OR pedestrian* OR (cycling N2 commut*) OR bike-riding OR ("activ* commut*") OR ("walk* N2 commut*"))) OR AB (("active transport*" OR "active travel*" OR "safe route*" OR pavement* OR sidewalk* OR "bicycle paths" OR (biking N2 commut*) OR "walking paths" OR "pedestrian crossing*" OR "traffic calming" OR "traffic lights" OR pedestrian* OR (cycling N2 commut*) OR bike-riding OR ("activ* commut*") OR ("walk* N2 commut*"))) OR MA (

("active transport*" OR "active travel*" OR "safe route*" OR pavement* OR sidewalk* OR "bicycle paths" OR (biking N2 commut*) OR "walking paths" OR "pedestrian crossing*" OR "traffic calming" OR "traffic lights" OR pedestrian* OR (cycling N2 commut*) OR bike-riding OR ("activ* commut*") OR ("walk* N2 commut*")))

7. TI (forests OR trees OR Park* OR recreation* OR "open space" OR "playing field*" OR "green space*" OR "open space*" OR "playing field*" OR "green space*" OR greenspace* OR greenspace* OR greenness OR greenery OR (recess N1 outdoor) OR schoolyard* OR "school garden*" OR "school ground*" OR "outdoor teach*" OR "outdoor learning" OR (nature N1 (acess OR contact OR recess)) OR "green schoolyard*" OR nature-based OR “forest school” OR “forest schools”)OR AB (forests OR trees OR Park* OR recreation* OR "open space" OR "playing field*" OR "green space*" OR "open space*" OR "playing field*" OR "green space*" OR greenspace* OR greenspace* OR greenness OR greenery OR (recess N1 outdoor) OR schoolyard* OR "school garden*" OR "school ground*" OR "outdoor teach*" OR "outdoor learning" OR (nature N1 (acess OR contact OR recess)) OR "green schoolyard*" OR nature-based OR “forest school” OR “forest schools”) OR MA (forests OR trees OR Park* OR recreation* OR "open space" OR "playing field*" OR "green space*" OR "open space*" OR "playing field*" OR "green space*" OR greenspace* OR greenspace* OR greenness OR greenery OR (recess N1 outdoor) OR schoolyard* OR "school garden*" OR "school ground*" OR "outdoor teach*" OR "outdoor learning" OR (nature N1 (acess OR contact OR recess)) OR "green schoolyard*" OR nature-based OR “forest school” OR “forest schools”)

8. (3 OR 4 OR 5 OR 6 OR 7)

9. 1 AND 2 AND 8

10. Limiters - Publication Type: Peer Reviewed Journal; English; Population Group: Human Published Year 2010-2022

Search strategy Transportation Services

Population

1. Population (“child*” OR “schoolchild*” OR “school aged child*” OR “school child*”)

Setting

2. Articles and papers and with language: English with keywords containing ("school" OR "schools" OR “primary school”* OR “school-based” OR “middle school*” OR "kindergarten" OR "pre school*")

Interventions

3. English with keywords containing “active travel” OR "active transport*" OR "safe route*" OR "pavement*" OR "sidewalk*" OR "bicycle paths" OR ("biking commut*") OR "walking paths" OR "pedestrian crossing*" OR "traffic calming" OR "traffic lights" OR "pedestrian*" OR ("cycling commut*") OR "bike-riding" OR "activ* commut*" OR (walk* AND ("commut*" OR "travel")))
4. Articles and papers and with language: English with keywords containing ("forests" OR "trees" OR "Park*" OR "recreation*" OR "open space" OR "playing field*" OR "open space*" OR "playing field*" OR "greenness" OR "greenery" OR “recess outdoor” OR "schoolyard*" OR "school garden*" OR "school ground*" OR "outdoor teach*" OR "outdoor learning" OR ("nature" AND (acess OR contact OR recess)) OR "forest school" OR "forest schools" OR "green schoolyard*" OR "nature-based")
5. Articles and papers and with language: English with keywords containing (“air pollut*” OR “air quality” OR “classroom ventilation” OR “coarse particle*” OR Soot OR “black smoke” OR “black carbon” OR “elemental carbon” OR “air clean*”)
6. Articles and papers and with language: English with keywords containing ("noise prevention") OR ("noise control") OR ("noise transport*") OR "noise exposure" OR "noise pollution" OR “road traffic noise” OR “traffic noise”
7. You searched with result type: Articles and papers and with language: English with title containing "environment design" OR "public facilities" OR "Urban Renewal" OR "City Planning" between dates 2010 – 2022 OR/ 3-7
8. 1 AND 2 AND 7

Search strategy Web of Science

Population

1. TI=(child* OR schoolchild* OR "school aged child*" OR "school child*")
2. AB=(schoolchild* OR "school aged child*" OR "school child*" OR "children" OR "child")
3. #1 OR #2

Setting

4. TI=(school* OR "primary school*" OR school-based OR "elementary school*" OR "middle school*" OR kindergarten OR "pre school*" OR preschool* OR pre-school*)
5. AB=("primary school*" OR school-based OR "elementary school*" OR "middle school*" OR kindergarten OR "pre school* " OR preschool* OR pre-school* OR school OR schools)
6. #4 OR #5

Intervention

7. TI=(built enviroment OR air pollut* OR "road traffic noise" OR "active travel*" OR "green spaces" OR "environment design" OR "public facilities" OR "Urban Renewal" OR "City Planning")
8. AB=("air quality" OR "classroom ventilation" OR "coarse particle*" OR soot OR "black smoke" OR "black carbon" OR "elemental carbon" OR "air clean*" OR "particulate matter" OR ("air pollution" NEAR/1 "indoor") OR ("air pollution" NEAR/1 "outdoor") OR ("noise" NEAR/1 "prevention") OR ("noise" NEAR/1 "control") OR ("noise" NEAR/1 "transport*") OR “transportation noise” OR "noise exposure" OR "noise pollution" OR "forests" OR "trees" OR "Park*" OR "recreation*" OR "open space" OR "playing field*" OR "open space*" OR "playing field*" OR "greenness" OR "greenery" OR (recess NEAR/1 outdoor) OR "schoolyard*" OR "school garden*" OR "school ground*" OR "outdoor teach*" OR "outdoor learning" OR ("nature" NEAR/1 (acess OR contact OR recess)) OR "forest school" OR "forest schools" OR "green schoolyard*" OR "nature-based" OR (Parks NEAR/1 recreation) OR “active travel” OR "active transport*" OR "safe route*" OR "pavement*" OR "sidewalk*" OR "bicycle paths" OR ("biking" NEAR/1 "commut*") OR "walking paths" OR "pedestrian crossing*" OR "traffic calming" OR "traffic lights" OR "pedestrian*" OR ("cycling" NEAR/1 "commut*") OR "bike-riding" OR "activ* commut*" OR (walk* NEAR/1 ("commut*" OR "travel")))
9. #7 OR #8
10. #3 AND #6 AND #9

Search strategy Scopus

Population

1. (TITLE-ABS (child*)) OR ((TITLE-ABS-KEY (schoolchild*)) OR (TITLE-ABS-KEY ("school aged child*")) OR (TITLE-ABS-KEY ("school aged child*")))

Setting

2. (TITLE-ABS-KEY (“school” OR schools OR "primary school*" OR "school-based" OR "elementary school*" OR "middle school*" OR "kindergarten" OR "pre-school*" OR "preschool"))

Interventions

3. TITLE-ABS-KEY ("environment design" OR "public facilities" OR "Urban Renewal" OR "City Planning" OR "air quality" OR "classroom ventilation" OR "coarse particle*" OR soot OR "black smoke" OR "black carbon" OR "elemental carbon" OR "air clean*" OR "particulate matter" OR ("air pollution" W/1 "indoor") OR ("air pollution" W/1 "outdoor") OR ("noise" W/1 "prevention") OR ("noise" W/1 "control") OR ("noise" W/1 "transport*") OR "noise exposure" OR "noise pollution" OR "forests" OR "trees" OR "Park*" OR "recreation*" OR "open space" OR "playing field*" OR "open space*" OR "playing field*" OR "greenness" OR "greenery" OR (recess W/1 outdoor) OR "schoolyard*" OR "school garden*" OR "school ground*" OR "outdoor teach*" OR "outdoor learning" OR ("nature" W/1 (acess OR contact OR recess)) OR "forest school" OR "forest schools" OR "green schoolyard*" OR "nature-based" OR “active travel” OR "active transport*" OR "safe route*" OR "pavement*" OR "sidewalk*" OR "bicycle paths" OR ("biking" W/1 "commut*") OR "walking paths" OR "pedestrian crossing*" OR "traffic calming" OR "traffic lights" OR "pedestrian*" OR ("cycling" W/1 "commut*") OR "bike-riding" OR "activ* commut*" OR (walk* W/1 ("commut*" OR "travel")))
4. #1 AND #2 AND #3
5. Limit to English, papers, published or in press, human

Search strategy Embase

Population

1. (child*.ti,ab OR schoolchild*.ti,ab OR "school aged child*".ti,ab OR "school child*".ti,ab)

Setting

2. (school*.ti,ab OR "primary school*".ti,ab OR school-based.ti,ab OR "elementary school*".ti,ab OR "middle school*".ti,ab OR kindergarten.ti,ab OR "pre school*".ti,ab OR preschool*.ti,ab OR pre-school*.ti,ab)

Intervention

3. ("active transport*".ti,ab OR "active travel*".ti,ab OR "safe route*".ti,ab OR pavement*.ti,ab OR sidewalk*.ti,ab OR "bicycle paths".ti,ab OR biking.ti,ab OR "walking paths".ti,ab OR "pedestrian crossing*".ti,ab OR "traffic calming".ti,ab OR "traffic lights".ti,ab OR pedestrian*.ti,ab OR cycling.ti,ab OR bike-riding.ti,ab OR commuting.ti,ab OR commute.ti,ab OR commuters.ti,ab)

4. ("air pollut*".ti,ab OR "air quality".ti,ab OR "classroom ventilation".ti,ab OR "coarse particle*".ti,ab OR Soot.ti,ab OR "black smoke".ti,ab OR "black carbon".ti,ab OR "elemental carbon".ti,ab OR "air clean*".ti,ab OR particulate matter.ti,ab)

5. ("road traffic nois*".ti,ab OR "traffic nois*".ti,ab OR "noise pollution".ti,ab OR "noise exposure".ti,ab OR "transport* noise".ti,ab OR (transport*.ti,ab ADJ2 Noise.ti,ab))

6. ("open space*".ti,ab OR "playing field*".ti,ab OR "green space*".ti,ab OR greenspace*.ti,ab OR greenspace*.ti,ab OR greenness.ti,ab OR greenery.ti,ab OR recess.ti,ab OR schoolyard*.ti,ab OR "school garden*".ti,ab OR "school ground*".ti,ab OR "outdoor teaching".ti,ab OR "outdoor learning".ti,ab OR (nature ADJ1 (acess OR contact OR recess)).ti,ab OR "green schoolyard*".ti,ab OR “forest school” .ti,ab OR “forest schools”.ti,ab OR nature-based.ti,ab OR forests.ti,ab OR forest.ti,ab OR (Parks ADJ1 recreation*))

7. (“Built Environment".ti OR "Urban Renewal".ti OR “City Planning”.ti)

8. 3 OR 4 OR 5 OR 6 OR 7
9. 1 AND 2 AND 8
