# Supplementary material for: School-Based Interventions to Support Healthy Indoor and Outdoor Environments for Children: A Systematic Review
Source: Int J Environ Res Public Health. 2023 Jan 18;20(3):1746. doi: 10.3390/ijerph20031746 (PMC9914556; doi:10.3390/ijerph20031746)
Supplement: Supplementary file 1 [file ijerph-20-01746-s001.zip › Supplementary Material S2 studies description.pdf]

Supplementary Material S2: Characteristics of included studies and effects of the interventions

Table S1: Characteristics of included studies: targeted intervention Air Pollution (n=10)

Brief name<br>*Author, year*	Participants, Characteristics, Country	Intervention	Outcome (Health, behaviors, and/or exposure levels only)	Main findings	Study design label and duration
Effects of air purifier on indoor air quality<br><br>(Oh et al., 2014)	7 childcare centers 2 classrooms with<br><br>13-20 children in each<br><br>5-7 years<br><br>Seoul, Korea	Air purifiers operation for 5 days	Indoor air quality<br><br>(PM2...5, PM10, bio-aerosols<br><br>airborne bacteria and fungi).	The removal efficiency ranged 58-85% for PM2.5, 49-86% for PM10, 41-68% for airborne bacteria, and 40 -68% for fungi depending on the school location (level of nearby traffic) and year of construction (>or < 15 years). The use of air purifiers may improve the indoor air quality through the exchange filters and internal controls in the system	Before-after<br><br>Duration: 3 weeks
Different ventilation methods and air purifiers for indoor pollutants<br><br>(Pacitto et al., 2020)	2 primary schools, NR<br><br>Barcelona, Spain	Four conditions: Natural ventilation, manual airing; natural ventilation and air purifiers; manual airing and air purifiers (Air change/hour: 1.7 h–1 to 9.2 h–1)	Indoor Air<br><br>Quality (Total particle number concentration, Black carbon, PM1-10, NO2, and CO2)	Use of air purifiers with windows kept closed reduced the indoor/outdoor concentration ratios of PM1-10 by 93-95%; in the larger school gym, the reduction was 70% and 84%.<br><br>For manual airing scenarios, the effect of the air purifiers on outdoor-generated sub-micron particles is reduced	Before-after<br><br>Duration: 4 weeks
Modifying the timing of the heating, ventilation, and air conditioning<br><br>(MacNeill et al., 2016)	4 schools<br><br>1 classroom each with<br><br>16-29 students (84 total)<br><br>Grade: 3-6<br><br>4-14 years<br><br>Ottawa, Canada	HVAC systems being started 1 h before rush hour and then turned off from the beginning of morning rush based on trends in nitrogen dioxide levels re-started	Indoor Air Quality<br><br>(Fine Particles, Black Carbon,<br><br>Ultrafine Particles,<br><br>Volatile Organic Compounds, CO2)	Adjusting the ventilation system to operate earlier in the morning, before high traffic commute periods significantly reduced traffic-related air pollutants within late-start schools (9 AM) located near major roads	Before-after<br><br>Duration: 32 days

Table: S1. (continued). Characteristics of included studies: targeted intervention Air Pollution (n=10)

**Brief name** ***Author, year***	**Participants, Characteristics, Country**	**Intervention**	**Outcome (Health, behaviors, and/or exposure levels only)**	**Main findings**	**Study design label and duration**
Mixing and displacement ventilation<br><br>(Smedje et al., 2011)	1 school, 4 classrooms,<br><br>111 pupils<br><br>Grade: 5th<br><br>Mean age 11.0<br><br>Uppsala, Sweden	New ventilation ducts were connected to the existing air supply ducts for mixing ventilation (4 weeks).<br><br>Comparison: Already installed displacement ventilation system (4 weeks)	Indoor air quality (airborne cat allergen CO2)<br><br>Respiratory symptoms: perception of the indoor environment, Health symptoms, and clinical signs of inflammation in the nose of pupils<br><br>(cat allergens, CO2)	69 pupils with completed data in both conditions.<br><br>CO2, humidity, and cat allergen were similar between ventilation modes.<br><br>Children perceived fewer respiratory symptoms ($p=0.054$) in Mixing ventilation conditions and eyes symptoms.<br><br>No significant difference in clinical signs.	Experimental cross-over<br><br>Duration: 8 weeks during wintertime
Effects of classroom ventilation on performance<br><br>(Bakó-Biró et al., 2012)	330-215 pupils from<br><br>16 classrooms of 8 schools<br><br>Grade: 5<br><br>9-10 years<br><br>Reading, England	Purpose-built mobile ventilation system installed in each classroom to control the ventilation rate temperature, provide outdoor air, or re-circulate the classroom air<br><br>Comparison: recirculated air	Cognitive performance<br><br>(CO2) - Viscope	Significantly faster and more accurate responses for Choice Reaction (by 2.2%), Colour Word Vigilance (by 2.7%), Picture Memory (by 8%), and Word Recognition (by 15%) at the higher ventilation rates (mean CO2 593-783 ppm) compared with the low ventilation (mean CO2 1638-4093 ppm) conditions.	Cross-over<br><br>repeated measures<br><br>Duration: 3 weeks across winter, summer, and spring
Effect of ventilation on air particulate matter<br><br>(Trompetter et al., 2018)	2 classrooms from one school, 27 children in each<br><br>7-9 years<br><br>Palmerston North,<br><br>New Zealand	Solar air heated ventilation unit for increasing classroom ventilation rates during winter<br><br>Comparison: unventilated classroom	Indoor Air Quality (PM2.5, PM10, indoor and outdoor)	Solar air heated ventilation unit reduced average CO2 concentration by 27% (from 1345 ppm to 980 ppm) and the average moisture level decreased by 12% (from 9.08g of water per kg of dry air to 7.98 g of water per kg of dry air). PM concentrations in the ventilated classroom were reduced to two-thirds of the concentrations measured in the unventilated classroom.	Controlled before-after<br><br>Duration: 3 weeks during winter season

Table: S1. (continued). Characteristics of included studies: targeted intervention Air Pollution (n=10)

Brief name<br>*Author, year*	Participants, Characteristics, Country	Intervention	Outcome (Health, behaviors, and/or exposure levels only)	Main findings	Study design label and duration
FRESH (Forced-ventilation Related Environmental School Health)	17 schools, 18 classrooms<br>18-36 pupils/school<br>Grade: 7th<br>10-11 years<br>The Netherlands	Mechanical ventilation device that was installed in classrooms to provide a stable ventilation flow with an adjustable outdoor air supply rate. Recirculation was used to ensure a steady airflow. Target CO2 concentrations were 800 and 1200 ppm.<br><br>Comparison: Control classrooms, natural ventilation continued according to the teachers' preference (n=6)			Longitudinal cross-over<br><br>Duration: 3 weeks during two heating seasons
(Rosbach et al., 2013)			Indoor Air Quality (CO2)	Significantly decrease CO2 levels in the intervention classrooms, with a mean decrease of 491 ppm. With the target set at 800 ppm, mean CO2 was 841 ppm (range: 743–925 ppm); with the target set at 1200 ppm, mean CO2 was 975 ppm (range: 887–1077 ppm).	
(Rosbach et al., 2016)			Indoor Air Quality (Endotoxin and β (1,3) -glucans, PM2.5, PM10, Indoor, and outdoor NO2)	Lowered endotoxin and β(1,3)-glucan levels and PM10 concentrations significantly. Concentrations of PM2.5 and NO2 were not affected by the intervention. In the control classrooms, however, the levels of all exposure variables increased somewhat.	

Table: S1. (continued). Characteristics of included studies: targeted intervention Air Pollution (n=10)

Brief name *Author, year*	Participants, Characteristics, Country	Intervention	Outcome (Health, behaviors, and/or exposure levels only)	Main findings	Study design label and duration
RaBe study<br>(Raumluftqualita¨t und das Befinden von Kindern–Indoor air quality and student experiences)<br>(Twardella et al., 2012)	6 schools, 20 classes<br>417 students<br>Grade: 3-4th<br>9-10 years<br>Bavaria and Berlin/ Brandenburg, Germany	Usual: The mechanical ventilation was adjusted as usual.<br>•Worse: The mechanical ventilation was down-regulated (CO_2 2000–2500 ppm)<br>•Better: The mechanical ventilation was up-regulated (CO_2 <1000 ppm.)	Cognitive performance - d2 test<br>(CO_2)	No significant effect of the experimental condition (median CO_2 level on average 1045 ppm) on concentration performance. However, the worse condition (median CO_2 average 2115 ppm) appears to decrease accuracy (1 to 1.5 points)	Cluster-randomized cross-over experimental<br>Duration: 3 weeks during wintertime
Mixing ventilation units with heat recovery<br>(Petersen et al., 2016)	2 schools, 2 classrooms each<br>70-78 pupils from both schools per test<br>Grade: 5th<br>10-12 years<br>Aarhus, Denmark	To lower the CO_2 concentration, single room mixing ventilation units with heat recovery were installed in each study classroom.<br>Comparison: recirculated air	Cognition (four numerical or language-based) and respiratory symptoms (18 visual analog scales)<br>(CO_2)	Correct answers were improved significantly: addition (6.3%), number comparison (4.8%), grammatical reasoning (3.2%), and reading and comprehension (7.4%) when the outdoor air supply rate was increased (from an average of 1,500 to 900 ppm), but only when "unmotivated" students' tests were discarded.<br>The intervention did not have any significant effect on the number of errors in any of the tests.<br>Pupils were experiencing less pain in the eyes in the recirculation condition compared to the fresh air condition.	Double-blind 2 x 2 crossover intervention<br>Duration: 3 weeks during Fall

Table: S1 (continued). Characteristics of included studies: targeted intervention Green Spaces (n=17)

**Brief name** ***Author, year***	**Participants, Characteristics, Country**	**Intervention**	**Outcome (Health, behaviors, and/or exposure levels only)**	**Main findings**	**Study design label and duration**
The Stephanie Alexander Kitchen Garden Program<br><br>(Block et al., 2012)	12 schools (6 control)<br><br>592 children (352 programs, 240 comparisons)<br><br>Grade: 3 to 6<br><br>8- 12 y<br><br>Victoria, Australia	The program model consists of all children participating, at a minimum, in a 45-minute garden class and a 1.5-hour kitchen class every week (mean 1.8 hours/week)<br><br>Comparison: No intervention	Child well-being; Child cooperative behaviors student attitudes<br><br>Robinson and Zajicek's scale (modified section)	Child wellbeing (p = .09). and cooperative behaviors (p= .7) demonstrated no statistically significant difference between mean scores at program and comparison schools in contradiction with qualitative data.	Quasi-experimental mixed methods<br><br>nonrandomized comparison<br><br>Duration: 12 to 25 months
Healthy Gardens, Healthy Youth<br><br>(Wells et al., 2014)	12 schools<br><br>227 (n=112 control) at baseline<br><br>Grades 4–5<br><br>8–12 y<br><br>New York, United States	The Garden was a 4′ × 8′ raised bed for each class. Access to a curriculum of 20 lessons for children; 11 lessons for year 1, and 9 for year 2. Aside from the lessons, educators led other activities in the garden such as planting, weeding, and harvesting.<br><br>Comparison: waitlist control group that received gardens at the end of the study	Physical Activity (Activity Questionnaire; Actigraph GT3X+ or GT1M; direct observation using PARAGON)	Data from accelerometry showed an increase of +.58 (p = .010); +1.0 (p = .044) in minutes of moderate and MVPA respectively compared to controls.<br><br>Direct observations within-group revealed that children move more and sit less during an outdoor garden-based lesson than during an indoor lesson.	Longitudinal cluster randomized controlled trial<br><br>Duration: 2 years
Texas!Grow!Eat!Go<br><br>TGEG<br><br>(Van Den Berg et al., 2020)	28 schools<br><br>Baseline 1326 students<br><br>Grade: Third<br><br>Texas, United States	School garden and a 32-lesson school curriculum that centered around the vegetables grown in the school gardens (TGEG) . Comparison: PA intervention (Walk Across Texas [WAT!]) only behavioral component, OR a combination of Garden and PA, OR neither Garden nor PA intervention	Physical Activity<br><br>(Marathon Kids Survey)	Main effects models indicated any of the school garden interventions did not increase physical activity. Within-group analyses show that compared to controls, children in the WAT! group significantly increased the number of times parents and children were active together (p = 0.038).	2x 2 factorial group randomized controlled trial<br><br>Duration: 6-months

Table: S1 (continued). Characteristics of included studies: targeted intervention Green Spaces (n=17)

**Brief name** ***Author, year***	**Participants, Characteristics, Country**	**Intervention**	**Outcome (Health, behaviors, and/or exposure levels only)**	**Main findings**	**Study design label and duration**
Outdoor lessons in nature<br><br>(Rees-Punia et al., 2017)	4 schools<br><br>75 children direct observation; 74 accelerometers portion<br><br>Grades: 2nd, 3rd, and 4th<br><br>Georgia, United States	One science or math lesson in the school garden each week during the study period (1,400 sq. ft to 14,100 sq. ft.; From average garden class time 15 to 45 minutes)<br><br>Comparison: within-subjects comparison of children's physical activity in garden and no garden school days	Physical activity<br><br>(Physical Activity Research and Assessment Tool for Garden Observation – PARAGON, ActiGraph GT3X)	Four schools averaged about ten additional minutes of MVPA on school garden days (accelerometers).<br><br>On average thirty-two minutes of sedentary classroom time was displaced with light physical activities in the garden among three of the four schools.	Observational within-subjects comparison<br><br>Duration: September through November (cross-over post only)
Outdoor lessons in nature<br><br>(Gustafsson et al., 2012)	2 elementary schools<br><br>230 children in both data collection<br><br>Grades: 0-6<br><br>6–11 y (8.4)<br><br>Linköping, Sweden	At least one hour each school day of outdoor teaching mostly takes place in the surrounding forest and woods. The main point of the intervention was to move the education from indoors to the natural green environment, and the school is located on the outskirts of a village in the countryside so has close access to forests for 6 months.<br><br>Comparison: reference school employed traditional methods; all education was book-based and consisted of paper-and-pencil work in the indoor classroom environment	Parent-version of Total difficulties score and subscales<br><br>(Strengths and Difficulties Questionnaire)	No evidence for a general effect of the intervention on total difficulties score and subscales. Significant differential effect on boys and girls; while boys at the intervention school generally displayed a decrease in mental health problems (coming from emotional symptoms, conduct problems and hyperactivity) compared to the reference school, the girls at the intervention school rather showed a non-change in mental health problems.	Quasi-experimental non-equivalent groups design<br><br>(nonrandomized control trial)<br><br>Duration.:1 year
Outdoor lessons in nature<br><br>(Largo-Wight et al., 2018)	1 school, 2 classes with<br><br>18 kindergartners each<br><br>5-6y<br><br>United States	The outdoor classroom was located in the school's play yard under a tree and shade tents. The space was defined with a circular boundary of tires, tree stumps, and potted plants.<br><br>Comparison: Indoor classroom	Child engagement behavior (teacher redirections) and child focus and attention (on-task behavior) direct observation frequency	For teachers' redirections of child behavior were significantly fewer in the nature condition (7.5 vs.10 redirections, $t=2.49$, $p < 0.05$) compared to the control, only for one of the two teachers' classrooms. No significant effects on attention.	Experimental crossover design<br><br>Duration: 6 weeks

Table: S1 (continued). Characteristics of included studies: targeted intervention Green Spaces (n=17)

**Brief name *Author, year***	**Participants, Characteristics, Country**	**Intervention**	**Outcome (Health, behaviors, and/or exposure levels only)**	**Main findings**	**Study design label and duration**
Outdoor lessons in nature<br><br>(Kuo et al., 2018)	Two classrooms (aprox. 32 students in total)<br><br>Grade: 3rd<br><br>9-10 y<br><br>United States	5-min walk from the classroom out to a grassy outdoor area with some nearby trees for a 30-min instructional period, followed by a walk back to the classroom, followed by a 5-min break<br><br>Comparison: the classroom lesson involved no walking, and a 40-min instructional period followed by a 5-min break.	Classroom Engagement - proxy of attention (Composite Index of Classroom Engagement.): teacher ratings, redirects, and independent photo-based<br><br>ratings	Out of 100 paired comparisons lessons, classroom engagement was over a full standard deviation better in the nature condition in 48 pairs; in 20 of those 48, the nature condition bested its classroom counterpart by over two standard deviations, rate of "redirects," was cut almost in half after a lesson in nature.	Mini experiment replicated<br><br>20 times (lesson nature vs same lesson in classroom)<br><br>Duration: 10 weeks (cross-over)
Outdoor lessons in nature<br><br>(Taylor and Butts-Wilmsmeyer, 2020)	Winter/spring: 3<br><br>Schools/ 2 classrooms/ 135 children<br><br>Fall: 6 schools/ 2 classrooms/250 children; Mean age at baseline: 5.0<br><br>Toronto, Canada	High Frequency of classes in nature: minimum 30 minutes (winter/spring) and 60 minutes (fall) of greenspace curriculum daily +total times outdoor (recess in schoolyards)<br><br>Comparison: Low frequency: 60-minute greenspace curriculum session per week	Behavioral self-regulation (Child Behavior Rating Scale - CBRS and Head-Toes-KneesShoulders Task HTKS task)	Higher frequency of greenspace curriculum was related to greater improvements in self-regulation, only significant for girls. More total time weekly in schoolyard green spaces was also related to higher self-regulation scores, only detected with the winter/spring, for both boys and girls, and fall only for girls.	Quasi-experimental design (controlled before- after)<br><br>Duration: 12 weeks winter/spring; 9 weeks fall
Urban forests / Forest<br><br>Therapy<br><br>(Bang et al., 2018)	5 community after-school centers<br><br>52 students (n= 28control); Grades: 4-6<br><br>Mean age: 11.83 exposed<br><br>Seoul, Korea	The 10-session health promotion program uses urban forests once a week. 30 min for the lecture and 60 min for the forest activities in the urban forests, which were 10 min away from each community center for children. Comparison: only the routine programs (e.g., supplementary learning) at their community center for children.	Peer relationships (self-report), and Attention deficit and hyperactivity (Conners-Wells Adolescents Self-Report Scales - CASS-S, short form)	No statistically significant changes were found for peer relationships and Attention deficit and hyperactivity between intervention and control groups.	Quasi-experimental control group pre-test–post-test design<br><br>Duration: 10 weeks

Table: S1 (continued). Characteristics of included studies: targeted intervention Green Spaces (n=17)

Brief name *Author, year*	Participants, Characteristics, Country	Intervention	Outcome (Health, behaviors, and/or exposure levels only)	Main findings	Study design label and duration
Wilderness Schooling<br><br>(Quibell et al., 2017)	9 schools,<br><br>Exposed: 223, Control: 217<br><br>8–11 years<br><br>United Kingdom	Visits to a National Trust site one day a week for six consecutive weeks (36 hours in all for each child). During the intervention days, children took part in several different tasks outdoors. Two days were science days, two English and two maths.<br><br>Comparison: curriculum delivered as normal by a teacher in the classroom	Educational attainment data in English reading, writing, and math (level scores)	Faster rate Improvement in the performance of the intervention group compared with that of the control group, respectively:<br><br>English reading: 4.30 (2.94) vs 1.39 (1.93)<br><br>Writing 2.70 (1.62) vs 1.37 (1.47)<br><br>Maths 3.45 (2.11) vs 2.21 (1.82)	Matched-groups design (controlled pre-post)<br><br>Duration: 6 weeks
Green breaks<br><br>(Amicone et al., 2018)	Study 1: 1 school, 82 children, 10.1 y<br>Study 2: 1 school, 36 children, 10.8 y<br><br>Rome, Italy	30 min break time recess time in the natural environment. Study 1: garden with 1,303 m2 and Study 2: natural area around 460 m2<br><br>Comparison: recess in-built environment. Study 1: courtyard in front of the school entrance (139 m2). Study 2: a built area around 460 m2 visible from the natural area and vice versa	Sustained and selective attention (Bells test), working memory (Digit Span), impulse control (go-no-go test)	Study 1: Sustained and selective attention (31.85 to 32.61, p= 0.016) and working memory (15.22 to 16.38, p <0.001) scores were higher after recess in nature. No significant effect was observed in built environment group, on impulse control in both groups.<br><br>Study 2: Increase of sustained and selective attention after (-0,18 vs. 0.37 - standardized means p = 0.007) the natural environment condition (vs. built) and a decrease after the built environment break	Study 1: a mixed-model crossover design.<br><br>Study 2: between-subjects quasi-experimental design<br><br>(Controlled pre-post)<br><br>Duration: two different weekdays

Table: S1 (continued). Characteristics of included studies: targeted intervention Green Spaces (n=17)

Brief name *Author, year*	Participants, Characteristics, Country	Intervention	Outcome (Health, behaviors, and/or exposure levels only)	Main findings	Study design label and duration
Nature-based Playground (Barton et al., 2015)	2 Primary schools<br>52 students<br>Year 5<br>Mean age:8.84±0.45<br>England	Recess in nature: 55-minute duration of lunch playtime on five consecutive days. Orienteering intervention was carried out on the school field and green areas surrounding the school buildings.<br>Comparison: lunchtime on playground sports consisted of small pieces of equipment such as skipping ropes, bats and balls and Frisbees	Physical Activity<br>(Actigraph GT1M)	The Playground sports increased MVPA (3.07±5.16 minutes) more than the recess in nature (2.15±4.31). Fitter children engaged more in MVPA in playground intervention (r=0.32; p<0.05), but not in the nature suggesting that this type of intervention may be effective at engaging children of all fitness levels.	Before-after cross-over<br>Duration: one week
School playing environment (Wood et al., 2014)	1 school<br>25 children<br>Mean age: 8.6±0.3<br>United Kingdom	Play on school field during morning (15 minutes) and lunchtime (one hour including the time taken to eat lunch) in the school field surrounded by trees and bushes.<br>Comparison: Play on the school playground during morning and lunchtime, which consisted of concrete areas surrounded by school buildings	Physical Activity<br>(Actigraph GT1M)	61.6% of the variance in MVPA was due to the playing environment and participants engaged in 40% more MVPA time when playing on the field (nature) compared to the playground. Girls engaged in an extra 59% MVPA during morning play, 72% during lunch play and 68% for all of playtime combined when playing in nature.	Counterbalanced, randomized cross-over design<br>Duration: 2 weeks/ 45 minutes of daily playtime
Playground greening project (Raney et al., 2019)	2 Title I elementary schools<br>437 students (355 experimental; 82 control) at least in one data collection<br>Grade:2nd,3rd, 4th, 5th<br>California, United States	~21,000 square feet of asphalt distributed in four playground zones were replaced by green space: trees, mulch, and boulders in two zones, with grass and trees; an outdoor classroom decomposed granite floor, mulch, and plant border, and log seating.<br>Comparison: a school that kept the asphalt playground	Physical activity (SOPLAY, and GT3X+); Social interactions (SOCARP);<br>Observing Play and Leisure; Activity in Youth (SOPLAY)	Vigorous activity participation increased pre to post at the individual (48.5%, 95% CI=29.1%, 67.9%, p<0.001) and population level (41.2%, 95% CI=27.3%, 55.1%, p=0.003) and remained higher than pre-greening at 4 months (p<0.05) for girls. Physical and verbal conflict rates decreased below pre-greening (3.5; CI=2.5, 4.5) rates after 4 months (1.8; CI=1.0, 2.6) for both sexes.	Nonrandomized, cluster-controlled trial<br>Duration: 4 months

Table: S1 (continued). Characteristics of included studies: targeted intervention Green Spaces (n=17)

**Brief name** ***Author, year***	**Participants, Characteristics, Country**	**Intervention**	**Outcome (Health, behaviors, and/or exposure levels only)**	**Main findings**	**Study design label and duration**
Schoolyard Greening (van Dijk-Wesselius et al., 2018)	9 elementary schools (4 controls) n= 706 children at baseline Age: 7-11 Grade: 4, 5 and 6 The Netherlands	Greening areas of schoolyards and also kept some areas paved. The green areas covert mostly features grassy hills, bushes, trees, tunnels made of tree branches, loose tree branches and garden-like parts<br>Comparison: 4 schools with paved schoolyards were mostly covered with tiles and contained some play equipment made of non-natural materials, like swings or climbing frames	Attention (Digit Letter Substitution test - DLST, Sky Search task - SST); Pro-social orientation (Social Orientation Choice Card – SOCC; peer problems, prosocial behavior from SDQ)<br>Physical Activity (ActiGraph GT3X)	Improved attention for both tasks after recess in green schoolyards was found at two-year follow-up. After greening children reported fewer peer problems and more social support.<br>Greened schoolyards stimulated girls' physical activity during a one-year follow-up.<br>Greening schoolyards seems especially beneficial for girls and younger children	Longitudinal prospective intervention study<br>Duration: 2 years
"Green Walls in Classrooms in Haarlemmermeer" (van den Berg et al., 2017)	2 elementary schools 170 children (n=86 control group) Mean age: 9.0 years Grades:5-7 Haarlemmermeer, The Netherlands	A "Wall so green" a single wall unit of 1.25 m wide and 2 m high was placed in the back of the room against the rear wall of one of the corners against a sidewall. The unit was stocked with eight types of green plants, including Spathiphyllum, Philodendron, and Dracaena<br>Comparison: classroom without green wall	Attention (Digit Letter Substitution test - DLST, Sky Search task - SST)<br>A five-item self-report measure of ability to concentrate	Green wall did not significantly influence the DLST scores (processing speed), neither as a main effect nor in interaction with time. Children in the classrooms with the green wall scored better than controls on (selective attention), it after controlling for baseline scores, grade level, and school, mean adjusted difference = 0.82, CI = [0.06, 1.57]. No significant differences for self-report concentration	Controlled prospective design (nonrandomized) Duration: 2 months
Greenery in the Classroom (Bernardo et al., 2021)	Study 1: 1One primary school, 4 classrooms, total sample 95 students (n=40 control group), 3rd grade<br>Study 2: Two schools, one classroom each, n=45 from middle-class area, n= 30 low-income area, 3rd grade Lisboa, Portugal	Two-steps intervention: (1) placement of an artificial green wall, 150 cm wide and 250 cm in height, in the classroom window area, (2) then an activity in which the children planted one lettuce in a pot with their teacher hen, each child was responsible for maintaining its lettuce, namely watering it and monitoring its growth. By the end of the experiment, each child had one lettuce, which they would harvest and take home	Selective and sustained attention (Bell test)<br>Working memory (Digital span test)	Study 1: A significant increase in sustained and selective attention (33 scores vs. 29.30, p = 0.000), and work memory (6.64 vs. 5.62 number of correct series p= 0.003) between the experimental and the control group, notably when vegetable pots were introduced. Study 2: In the second moment (green walls), only the work memory showed a significant effect tested with the inversed number of the Digital Span test (6.64 vs 5.60 number of correct series p= 0.002)	Study 1: controlled before-after<br>Study 2: before-after<br>2 months

Table: S1 (continued). Characteristics of included studies: targeted intervention Active travel (n=12)

**Brief name** ***Author, year***	**Participants, Characteristics, Country**	**Intervention**	**Outcome (Health, behaviors, and/or exposure levels only)**	**Main findings**	**Study design label and duration**
Safe Routes to School (SRTS) program<br><br>(McDonald et al., 2013)	9 primary schools and 5 middle schools (includes 9 experimental and 5 control schools)<br><br>~1000–2300 students<br><br>school year<br><br>Grade: K8<br><br>Eugene, Oregon, United States	4E's: engineering, education, encouragement, and enforcement. Engineering interventions are usually infrastructure improvements, such as side-walk construction, crosswalks, and traffic signal improvements.	Proportion of students walking, and biking combined (Hands-up classroom survey and parents' survey)	• Improving sidewalks and crosswalks had a non-significant impact (p=0.058) on walking and biking, however, improvements were completed late in study period.<br><br>• Augmenting education programs with additional improvements (i.e., covered bike parking) was associated with increases in walking and biking of 5–20 % points<br><br>• Grade-level strongly predicted walking (5th to 8th), but not biking	Quasi-experimental<br><br>(nonrandomized)<br><br>Duration: up to 4 years
(McDonald et al., 2014)	801 schools (>80% elementary schools), 4,504 unique observations of school travel mode by school<br><br>K8<br><br>Florida, Oregon, Texas,<br><br>District of Columbia	Comparison: schools without SRTS program		Adjusted models by school and neighborhood characteristics, walking and bicycling rose by 1.1 percentage points (p = .002) with each year of participation in the SRTS program. Engineering improvements are associated with an 18% relative increase in walking and bicycling.	Controlled before-and-after<br><br>Duration: 5 years

Table: S1 (continued). Characteristics of included studies: targeted intervention Active travel (n=12)

**Brief name *Author, year***	**Participants, Characteristics, Country**	**Intervention**	**Outcome (Health, behaviors, and/or exposure levels only)**	**Main findings**	**Study design label and duration**
Safe Routes to School (SRTS) programs (Stewart et al., 2014)	53 schools<br><br>79% primary school*<br><br>Alaska, Florida, Mississippi, Texas, Wisconsin, and Washington.<br><br>United States<br><br>*Focus on K8 (5 to 14y) even though the specific age was not reported	Noninfrastructure activities and infrastructure interventions:<br><br>sidewalk or crosswalk construction, installation of permanent<br><br>signage, Americans with Disabilities Act improvements such as curb cuts, bicycle rack installation, traffic calming/control such as speed bumps, construction of a shared-use path,<br><br>bicycle lane installation, and construction of pedestrian overpasses or bridges.	Changes in rates of walking, and bicycling: in-class tallies from grant applications, closing project reports, and direct coordinator requests or site visits	Statistically significant increases in active travel were observed across projects in all four states. At school level, all active modes increased from 12.8% to 19.8% ($p < .001$); walking from 8.8% to 13.3%; and bicycling from 2.0% to 3.2% ($p = 0.085$). Increases in rates of bicycling were negatively correlated with baseline rates of bicycling.	Before-after<br><br>Duration: 5 years
T-COPPE Texas Childhood Obesity Prevention Policy Evaluation (Hoelscher et al., 2016)	78 elementary schools<br><br>Baseline: 3,315 children<br><br>Grade: 4th<br><br>Texas, United States	A federally funded program designed to increase active travel to school through infrastructure and non-infrastructure strategies., which included engineering project(s) such as sidewalks, crosswalks, and so forth.<br><br>Schools awarded infrastructure projects<br><br>(n = 23), schools awarded non-infrastructure projects (n = 21)<br><br>Comparison: matched schools with no funding (n = 34)	Walking or cycling to school counts obtained by student self-report for before school (morning) and after school (afternoon) commutes in class;<br><br>Physical activity: number of days of 60'; number days of 30 'outdoor; number of days participated in sports lessons from children and their parents' cross-sectional surveys	Morning percent active commuting to school in infrastructure projects and non-Infrastructure projects were higher than in comparison schools ($p = .024$, $p = .013$, respectively).<br><br>Students from all groups reported more days spent taking lessons or practicing sports during the last week ($p < .001$). Students from non-infrastructure and comparison schools reported more days with 30 min or more of daily outdoor physical activity ($p < .05$).<br><br>Modest effects with decrease over time<br><br>Non-infrastructure funding appears to have slightly negative effects on active travel over time	Quasi-experimental using a serial cross-sectional sample<br><br>pretreatment/posttreatment design<br><br>Duration: 3 years after

Table: S1 (continued). Characteristics of included studies: targeted intervention Active travel (n=12)

**Brief name *Author, year***	**Participants, Characteristics, Country**	**Intervention**	**Outcome (Health, behaviors, and/or exposure levels only)**	**Main findings**	**Study design label and duration**
School Travel Plan (STP)<br><br>(Hinckson and Badland, 2011)	Auckland, New Zealand<br><br>33 elementary schools<br><br>13,631 students	A combination of engineering, education, enforcement, encouragement, and policy strategies. Changes to the infrastructural environment through implementation of crossings, cycling facilities, speed-reduction devices, road markings, and signage	Hands-up surveys to assess child travel modalities to and from school (walking, walking school bus, and cycling modes)	Percentage point changes in travel modes, with active transport increasing by 5.9%±6.8% when compared to baseline travel modes. School roll, year of implementation, and baseline values predicted engagement with active transport	pre-post<br><br>Duration: 12 to 24 months
(Hinckson et al., 2011)	56 primary schools<br><br>57,096 children<br><br>Grade: 0-5<br><br>Age: 5-10			Intervention increased active travel from 40.5% to 42.2% (OR=2.65) after 3 years of implementation in small, medium, and large schools. This increase was higher in students of high socioeconomic, in smaller schools, and in older children ($p < 0.05$)	pre-post<br><br>Duration: 3 years
SR2S California Safe Routes to School Program<br><br>(Ragland et al., 2014)	8 schools,<br><br>1,999 caregiver's surveys<br><br>Grades: 3-5<br><br>California, United States	Engineering modifications such as sidewalks, crosswalk placement and painting, traffic lights, and speed humps near schools make it safer for children to walk or bicycle	Mode shift to walk<br><br>(Parent's Survey)	The analysis found that living within 250 ft of an SR2S project increased the probability that a child walked to school (coefficient = 0.82, Z-statistic = 2)	Pre-post evaluation<br><br>Duration: 10 years

Table: S1 (continued). Characteristics of included studies: targeted intervention Active travel (n=12)

**Brief name *Author, year***	**Participants, Characteristics, Country**	**Intervention**	**Outcome (Health, behaviors, and/or exposure levels only)**	**Main findings**	**Study design label and duration**
School travel plan (STP)<br><br>(Buliung et al., 2011)	12 schools<br>1489 children<br>Mean age: 8.5<br>Duration:<br>Alberta, Nova Scotia, Ontario, British Columbia. Canada	Interventions were classified using 4 categories: (1)education, (2) activities and events, (3) capital improvement projects, and enforcement (varied between schools).<br><br>Capital improvements included removal/trimming of shrubs around school, improvement of walking route in the immediate area around the school, installing 4-way stop, signage, yellow school zone, no parking, sidewalk repair, and more.	Percentage of students walking, bicycling, or using any active mode: Hands-up classroom surveys; Household questionnaires reported by parents (all active modes combined)	Education was the most widely implemented type of intervention (38.0%). The rate of active transportation across all schools increased from 43.8% (baseline) to 45.9% (follow-up), d = 0.05. Based on the household survey at follow-up, the overall rate of active travel at follow-up was slightly different: 37.3% to school and 43.5% from school.	Before-after<br><br>Duration: 1 year
"Tryg og Sikker Skolecykling"<br><br>(Safe and secure cycling to school)<br><br>(Ostergaard et al., 2015)	25 primary schools<br>(12 control)<br>Baseline: 2,401 children<br>Average 11 years at baseline<br>Grade:4th and 5th<br><br>Copenhagen - Denmark	"Hard" interventions included structural changes near the school e.g., road surface, signposting, and traffic regulation such as one-way streets and regulation of car drop-off zones. Comparison: schools not involved in any physical activity promotion projects during the study period	Anthropometry; Cardiorespiratory fitness Physical activity beyond transport to school and Physical activity from cycling; Active travel to school	Cardiorespiratory fitness decreased in the intervention group relative to the control group (B = −1.45 ml O2·kg−1·min−1; p < 0.0001). No change in recreational cycling, overall, physical activity, BMI and obesity. Change in the number of cycling trips to/from school were not significant (B = 0.15 trips; 95% CI = −0.25; 0.54) d = 0.02.	Quasi-experimental<br><br>Duration: 1 year<br><br>(nonrandomized)
Changing Road Infrastructure/ Safer Communities programme (Smith et al., 2020)	2 schools, Baseline: 123, Follow-up: 152 children<br>Year 5 and 8<br>~Age:8-13<br>New Zealand	Small to medium scale school-focused infrastructure to improve pedestrian safety. Specific infrastructure treatments included: pedestrian refuge added to existing median barrier, path upgrade, and removal of corner barrier to improve safe	Active travel to school: Parents' and children's questionnaires; Routine tube count and video cameras	Findings showed no significant increase in active school travel after the intervention. Rather, a reduction in walking for the school journey was observed, with a concomitant increase in car travel	Before-after<br><br>Duration: 1 year

Table: S1 (continued). Characteristics of included studies: targeted intervention Active travel (n=12)

**Brief name** ***Author, year***	**Participants, Characteristics, Country**	**Intervention**	**Outcome (Health, behaviors, and/or exposure levels only)**	**Main findings**	**Study design label and duration**
School Travel Plan (STP)<br><br>(Mammen et al., 2014a)	Canada<br><br>53 elementary schools<br><br>Age: 6-14	School travel planning is a multi-disciplinary, multi-sectoral, school-specific intervention. Types of strategies typically emerging from action plans: educational strategies (e.g., hosting educational workshops to promote the awareness and benefits of AST); activities and events (e.g., organizing AST supervision via walking school bus schemes); capital improvement projects (e.g., installing a sidewalk or bike rack); and enforcement initiatives (e.g., increased police presence or crossing guards).	Active travel to school: Hand's Up survey	At the national level, there was no significant increase in active travel after one year. Baseline and follow-up data showed that 27% and 31% of children engaged in active travel to and from school, respectively. Active travel ranged post-intervention from a decline of 26% to an increase of 23% across schools.	Before-after<br><br>Duration: 1 year
(Mammen et al., 2014b)	7827 questionnaires from parents of children attending schools with STP program<br><br>Grade: K-8		Proportion of students who switched from driving to active travel after one year of baseline (parents' survey)	17% of the sample reported driving less at one-year follow-up both in the morning and afternoon periods – 83% of this total changed from driving to active travel.	Cross-sectional retrospectively post-intervention<br><br>Duration: 1 year

BMI = Body mass index; HVAC = Heating, Ventilating, and Air Conditioning; CO_2 – carbon dioxide; NO_2 - Nitrogen dioxide; O_3 -Ozone; $PM_{2.5}$ - particulate matter ≤ 2.5 micrometers; PM_{10} - particulate matter ≤ 10 micrometers; MVPA = Moderate to Vigorous Physical Activity; NR = not reported
