# Supplementary material for: School-Based Interventions to Support Healthy Indoor and Outdoor Environments for Children: A Systematic Review
Source: Int J Environ Res Public Health. 2023 Jan 18;20(3):1746. doi: 10.3390/ijerph20031746 (PMC9914556; doi:10.3390/ijerph20031746)
Supplement: Supplementary file 1 [file ijerph-20-01746-s001.zip › Supplementary Material S3 TiDIER description of included studies.pdf]

## Supplementary Material S3: TIDieR analysis of the interventions. The Template for Intervention Description and Replication (TIDieR) a standardised checklist and guide commonly used to analyse health interventions (n=39)

| BRIEF NAME[1] | WHY[2] | WHAT (MATERIALS)[3] | WHO PROVIDED[4] | HOW[5] | WHERE[6] | WHEN and HOW MUCH[7] | TAILORING[8] | MODIFICATIONS[9] | HOW WELL[10] |
|---|---|---|---|---|---|---|---|---|---|
| Effects of air purifier operation on indoor air quality in childcare centers<br><br>(Oh et al., 2014) | The use of an air purifier may be considered in cases where natural ventilation is used but concerns exist of indoor pollution from external sources, or if the ventilation system is not effective at removing particles | Air purifier (LA-R119SWF, Korea) | Researchers (funded by Korea Ministry of Environment) | The air purifier was operated for five days in two classrooms (18-21 children)). The concentrations of particulate matter and bio-aerosols were analyzed during twenty days, and were compared before and after air purifier operation. | Two classrooms from 7 childcarers located in Seoul Korea residential childcare centers which were near apartments and houses, childcare centers in commercial areas located near roadways adjacent to heavy traffic areas, and childcare centers existing near construction sites in Korea | During the summer (6/29/2013 and 8/ 31/2013), fall (9/1/2013 and 11/30/2013 ) and winter (1/6/2014 and 2/21/ 2014) periods over three weeks | No | Not stated | Not stated |
| Different ventilation methods and air purifiers on indoor pollutants<br><br>(Pacitto et al., 2020) | School gyms are present high $PM_{10}$ concentrations mainly due to the resuspension phenomena occurring during the physical activity itself. No effective reduction strategies are typically adopted in gyms since the ventilation relies upon natural ventilation and manual airing | Air purifier had maximum flow rate of 660 m3 h−1, dimensions of 60 × 73 × 55 cm, power of 178 W, electrical intensity of 1.1 A. Regarding filtration characteristics, the purifiers adopted i) pre-filtration with a glass microfiber filter class F7 for particles with a diameter b 0.2 μm; ii) a filter for gaseous pollutants containing a mixture of active carbon and absorbent media able to filter H2S, SO2, NO, NO2, HCHO, as well as hydrocarbons, chlorine, and volatile organic compounds; iii) a glass microfiber HEPA filter H14 for submicron and ultrafine particles. The six purifiers used at the maximum power provided a total flow rate of 3960 m3 h−1. | Researchers (not funding reported) | One week of measurements for each strategy in each school gym:<br><br>• natural ventilation - windows kept closed during the schooltime (ventilation just due to the leakages of the envelope)<br>• manual airing- windows open during the school time<br>• natural ventilation and air purifiers - windows kept closed<br>• natural ventilation and air purifiers<br>•manual airing and air purifiers | 2 public primary school gyms in the urban area of Barcelona (Spain). Both school gyms present a rectangular floor shape 430 and 2400 m3 for gym A and B, respectively. Hot-water plants are installed in the gyms no Adhoc mechanical ventilation systems were present. Air ventilation only relied upon leakages of the envelope (natural ventilation) or window opening (manual airing) | February–April 2015 for four weeks in each school gym. | No | Not stated | Not stated |

| BRIEF NAME[1] | WHY[2] | WHAT (MATERIALS)[3] | WHO PROVIDED[4] | HOW[5] | WHERE[6] | WHEN and HOW MUCH[7] | TAILORING[8] | MODIFICATIONS[9] | HOW WELL[10] |
|---|---|---|---|---|---|---|---|---|---|
| Modifying the timing of the heating, ventilation, and air conditioning (HVAC)<br><br>(MacNeill et al., 2016) | Less attention is placed on reducing population exposure to traffic sources through land-use policies, or building/ventilation practices. This strategy could be readily implemented for buildings with programmable HVAC system | The intervention comprised of the HVAC systems being started prior to rush hour traffic based on daily trends in nitrogen dioxide levels. HVAC systems in the Ottawa schools were re-started 1 h prior to rush hour and then turned off from the beginning of morning rush hour until the systems were required to be operational for occupation of the building. | Health Canada, in collaboration with the Ottawa-Carleton District School Board (funded by Clean Air Regulatory Agenda) | For all schools, the HVAC system was taken out of nighttime mode to flush the building air between 5:30 and 6:30 AM, followed by a period of recirculation until school began. As two of the schools were early (8:00 AM)-start schools and two others late (9:00 AM)-start schools, they resumed normal ventilation programming based on their start times; early-start schools resumed ventilation at 8:00 AM, while late-start schools resumed ventilation at 9:00 AM | Four Ottawa public elementary schools: two schools were early-start schools (8 AM–2:30 PM), and two schools were late-start schools (9 AM–3:30 PM). Measurements were collected from three locations at each school: (i) in one classroom; (ii) outdoors near the HVAC air intake; and (iii) outdoors near the front door. | In October and November of 2013, a total of 16 consecutive school days of baseline monitoring and 16 consecutive school days of intervention monitoring were completed at each school. | No | Not stated | Not stated |
| Mixing and displacement ventilation<br><br>(Smedje et al., 2011) | Displacement ventilation (DV) was related to fewer respiratory symptoms in pupils compared to mixed flow ventilation (MV). It is unclear whether these results were because of the ventilation principle or because of the fact that the displacement systems were new, clean, and had higher ventilation rates | In each classroom, there were two semicircular supply diffusers for DV (Halton ABF200-1200), both located at the front of the room, and exhaust air terminals on the ceiling level on the opposite side of the room. New ventilation ducts were connected to the existing air supply ducts, and two square diffusers for MV (Polman P01b) were mounted at the ceiling level so that it was possible to switch between DV and MV keep the ventilation rate constant. The supply air was filtered through F7 (=EU7) filters | Researchers (funded by Swedish research council FORMAS grant) | Each displacement diffuser had a totally free supply area of 997 $cm^2$ and a perforation degree of 9.25%. Corresponding values for the mixing diffusers were 314 $cm^2$ and 12.5%, respectively. In two classrooms, one on each floor, DV was applied for a period of 4 weeks, while it was MV in the other two classrooms. After 4 weeks, the alternative ventilation principle was applied for a further 4-week period. | In four classrooms in one school building in Uppsala, Sweden | During wintertime (February and March) over 8 weeks period. | No | Not stated | When comparing DV and MV in the field, it is important to recognize that the intended operation may not always be fully obtained. |

| BRIEF NAME[1] | WHY[2] | WHAT (MATERIALS)[3] | WHO PROVIDED[4] | HOW[5] | WHERE[6] | WHEN and HOW MUCH[7] | TAILORING[8] | MODIFICATIONS[9] | HOW WELL[10] |
|---|---|---|---|---|---|---|---|---|---|
| Effects of classroom ventilation on pupils' performance<br><br>(Bako-Biro 2012) | Indoor air quality in classrooms by using $CO^2$ as an indicator of ventilation and shows how it affects the performance of mental tasks using in-situ direct measurements | Built mobile ventilation system to control the ventilation rate in classrooms. The system was set either to provide outdoor air or to re-circulate the classroom air. The ventilation system consisted of an exterior fan placed outdoors, ductwork with a diameter of 200 mm supplied the air into the building through window openings, which were covered with Perspex plates and cut to connect to the ducts. the air was distributed using Softflo air terminal units, which consist of a perforated duct with small nozzles creating confluent jets flowing into the room. Sound attenuators were installed. | Researchers (funded by Cleaning Industry Research Institute International) | Provision of fresh air/recirculated air conditions was made in a cross-over repeated-measures design for the two classrooms. The order of presentation of the two ventilation conditions in weeks two and three was balanced. The capacity of the supply fan was selected to provide 200 l/s, matching the prescribed level of 8 l/s per person in a classroom holding, on average, 25 children. | Eight schools in two classrooms in England each over different seasons. The classrooms were all similar in size and construction | February 2006 to 2008 during two consecutive weeks. During winter (2 schools), spring (2 schools), early summer (2 schools) and autumn (2 schools). | No | Not stated | The outdoor measurements at some schools were not available due to technical failures. |
| Effect of ventilation on air particulate matter<br><br>(Trompetter et al., 2018) | There is a need for more accurate methods to disentangle source contributions in the indoor environment and to more clearly elucidate potential health effects | A prototype solar air heater was made of a double-layer roof (a polycarbonate layer over a color steel corrugated north-facing roof, where outdoor air was passed between the two layers). From front to back, the unit (SolarVenti™ SV30 model) consists of a polycarbonate cover (solar radiations fall on this cover), an absorber made of black felt and a transpired aluminum plate. | Researchers (Funded by Housing and Health Research Group/He Kainga Oranga, Health Research Council of New Zealand, Lottery Grants Board, SolarVenti Australia and GNS Science Direct Core Funding.) | The ambient air enters the unit from the back (hundreds of tiny holes in the transpired plate), circulates thru the absorber (temperature gain) and exits the unit from an outlet located at the end of the unit. A 12-volt photovoltaic panel powered a fan that pushes warm air into the classroom. The unit provided an average air flow of 1.8 m s−1 (SD = 0.37 m s−1) which gives an estimated ventilation rate of 82 ± 16 m3 h−1 during the study period while the classroom was occupied (08:45–15:00) | Two classrooms from a primary school in Palmerston North New Zealand. Control and intervention classrooms were of 1960s building stock and had synthetic fiber carpet floor coverings | August 2013 (winter season in New Zealand) over 3 weeks | No | Not stated | The average temperature was 1.9 °C higher in the control classroom than in the treatment classroom. This result was not expected. The teacher in the control classroom operated the heater 3x times more than treatment classroom |

| BRIEF NAME1 | WHY2 | WHAT (MATERIALS) 3 | WHO PROVIDED4 | HOW5 | WHERE6 | WHEN and HOW MUCH7 | TAILORING8 | MODIFICATIONS9 | HOW WELL10 |
|---|---|---|---|---|---|---|---|---|---|
| FRESH (Forced-ventilation Related Environmental School Health) | There is still a need for more experimental evidence with respect to the relationship between classroom ventilation and its effect on both respiratory health and cognitive performance. Focus on the performance of the ventilation system in terms of achieved classroom $CO_2$ concentrations. | Mobile custom-made mechanical ventilation device providing a stable ventilation flow with an adjustable outdoor air supply rate. The device consisted of an exterior constant flow fan (LAAHP12) placed outdoors. Within the device outdoor air was mixed with indoor air derived from the classroom via the return system. The device was CO2 controlled, using a real-time, self-calibrating CO2 sensor (Telair 6613) to adjust the amount of outdoor air supplied, in order to achieve a target steady-state CO2 concentration in the classroom | Researchers (funded by The Netherlands Organisation for Health Research and Development) | The mixture of indoor and outdoor air was heated before being introduced into the classroom with a flow of approximately 1400 $m^3$/h. Simple ducting (diameter 355 mm) lead the air without filtering into the building through a tailor-made window pane. In the classrooms, the air was distributed through a flexible, perforated fabric air sock. A non-flexible duct was used for air exhaust. The first week served as baseline period. In the following two weeks, in the intervention classrooms the concentrations of CO2 were maintained at pre-set levels of 800 and 1200 ppm | 12 primary intervention schools' classrooms and six controls located in two regions in the north and north-eastern parts of The Netherlands with comparatively low concentrations of ambient air pollutants (Zwolle and Groningen, The Netherlands) | Heating seasons (October-April) of 2010–2011 and 2011–2012 over three weeks. | No | Not stated | Not stated |
| (Rosbach et al., 2013) | | | | | | | No | No | Due to various causes (such as failing equipment, not all indoor air measurements were available for all classrooms. |
| (Rosbach et al., 2016) | Focus on increase in outdoor air pollutants within the classroom | | | | | | | | |

| BRIEF NAME[1] | WHY[2] | WHAT (MATERIALS)[3] | WHO PROVIDED[4] | HOW[5] | WHERE[6] | WHEN and HOW MUCH[7] | TAILORING[8] | MODIFICATIONS[9] | HOW WELL[10] |
|---|---|---|---|---|---|---|---|---|---|
| RaBe study (Raumluftqualitat und das Befinden von Kindern – Indoor air quality and student experiences)<br><br>(Twardella et al., 2012) | The relevance of air quality as indicated by $CO_2$ level in the classroom for the attention and concentration of the students could not be consistently shown yet | Usual: The mechanical ventilation was adjusted as usual.<br><br>Worse: The mechanical ventilation was down-regulated (CO2 2000–2500 ppm)<br><br>Better: The mechanical ventilation was up-regulated (CO2 <1000 ppm.) | Researchers (Funded by the Deutsche BundesstiftungUmwelt (DBU)) | The beginning of the first-class hour until the end of the cognitive test in the fourth-class hour (not counting breaks) thus resulted in an exposure duration of typically, 155 min. Experimental conditions were implemented 2 days per week. | Two to six classes from six primary schools five of which were located in the State of Bavaria and one close to Berlin. | November 2009 and April 2010 over three consecutive weeks. | No | Not stated | Not able to achieve the exact CO2 levels as planned by regulation of the mechanical ventilation system. |
| Mixing ventilation units with heat recovery<br><br>(Petersen et al., 2016) | Increased outdoor air supply rate has a positive effect on the performance of schoolwork by children | Single-room mixing ventilation units with heat recovery. The setting of the ventilation distribution grill in the ventilation units was customized for each study classroom. The ventilation units in school A were standing on the floor in the back corner. The ventilation units in school A were standing on the floor in the back corner. The ventilation units in school B were wall-mounted units placed on the facade at the ceiling. The ventilation units in school B were wall-mounted units placed on the facade at the ceiling. Ventilation units were equipped with an electrical heating coil with a set point of 22°C. | Researchers<br><br>(AirMasterA/S for providing the ventilation units) | To lower the CO2 concentration, single room mixing ventilation units with heat recovery were installed in each study classroom. In the 'fresh air' intervention condition, the ventilation units were set to run at a variable air volume controlled by a maximum CO2 concentration of 1,000 ppm measured in the ventilation outlet. In the 'recirculation' intervention condition, the air intake was blinded off, but the fans were still running generating the same level of noise as in the 'fresh air' intervention condition according to manufacturer's specifications. | Two classrooms from two 70s schools near Aarhus, Denmark. The ventilation principle in both schools is single-sided natural ventilation. Heating is provided with water-filled radiators. The classrooms have typical school furniture, the floor is covered with linoleum, and the wardrobe is situated just outside the classroom | In the fall of 2013 (16 September to 4 October) which, in the Danish climate, is a transitional period between non-heating and heating seasons over 3 weeks | No | Not stated | Not stated |

| BRIEF NAME[1] | WHY[2] | WHAT[3] | WHO PROVIDED[4] | HOW[5] | WHERE[6] | WHEN and HOW MUCH[7] | TAILORING[8] | MODIFICATIONS[9] | HOW WELL[10] |
|---|---|---|---|---|---|---|---|---|---|
| The Stephanie Alexander Kitchen Garden Program (SAKG)<br><br>(Block et al., 2012) | School gardening and garden-based nutrition programs have the potential to improve children's nutritional and health status as well as have a positive impact on social and environmental behavior. However, there remains a lack of rigorous peer-reviewed research providing sufficient evidence for their outcomes | The SAKG Program provides a seed-to-table experience, offering the opportunity to plant, nurture, harvest, prepare, and share fresh, nutritious, and seasonal food. Children keep kitchen garden program journals. Children are actively involved in all aspects, from garden design, preparing beds, planting seeds, transplanting seedlings, nurturing the growing plants. In the kitchen, children work in small groups to prepare sophisticated, multi-course meals based on seasonal produce from the garden<br><br>Core elements of the program are set out in a program manual: https:// www.kitchengardenfoundation.org.au/ | Garden and kitchen classes are planned and supervised by specialist staff assisted by the classroom teacher and adult volunteers. In most, although not all cases, specialist staff are employed who have prior qualifications and experience in horticulture and hospitality, respectively<br><br>(Funded by the Stephanie Alexander Kitchen Garden Foundation) | All children in Grades 3 to 6 (aged 8-12 years) participate, at a minimum, in a 45-minute garden class and a 1.5-hour kitchen class every week as an ongoing part of the school curriculum | Garden and kitchen classes at school (not detailed) of 6 schools in Victoria, Australia | Local adaptations by schools of the program model resulted in a range of exposure to program activities from 1.25 hours to 2.5 hours per week (mean 1.8 hours) for each student, over 12 to 25 months. | Schools are able to adapt weekly program activities to suit their particular social and physical environments and constraints as well as to respond to local events and other aspects of the school curriculum | Local adaptations by schools of the program model resulted in a range of exposure to program activities from 1.25 hours to 2.5 hours per week (mean 1.8 hours) for each student | Not stated |
| Healthy Gardens, Healthy Youth<br><br>(Wells et al., 2014) | For evidence-based garden interventions to be developed and implemented, there is a need for a clearer understanding of the potential for gardens to bolster children's physical activity and reduce sedentary behaviors. | The intervention consisted of four components: (1) the garden was a 4′ × 8′ raised bed for each class, and (2) an educational toolkit containing lessons focused on nutrition, horticulture, and plant science. (3) Resources for the school included information about food safety in the garden and related topics. (4) The garden implementation guide provided guidance regarding planning, planting and maintaining the garden throughout the year; gardening during the summer; engaging volunteers; building community capacity; and sustaining the program. https://peoplesgarden.wsu.edu/ toolkit/ | Lessons are led either by the classroom teacher or a Cooperative Extension Educator as part of Healthy Gardens, Healthy Youth pilot program<br><br>(Funded by Robert Wood Johnson Foundation, U.S. Department of Agriculture) | Schools were randomly assigned to the garden intervention or to the waitlist control group that received gardens at the end of the study. A garden was a 4′ × 8′ raised bed for each class. Access to a curriculum of 20 lessons for children in grades 4–6; 11 lessons for year 1, and 9 for year 2. Aside from the lessons, educators led other activities in the garden such as planting, weeding, and harvesting. | 12 schools in 5 regions of New York State within rural, urban, semi-urban areas. | Baseline data were collected in Fall 2011. The garden intervention began in Spring 2012 and continued through Spring 2013. Three waves of post-garden implementation data were collected (wave 2: late Spring 2012, wave 3: Fall 2012, wave 4: late Spring 2013) | No | Not stated | Moreover, this article does not examine the fidelity of the garden intervention, which is likely to differ from one school to another. |

| BRIEF NAME[1] | WHY[2] | WHAT[3] | WHO PROVIDED[4] | HOW[5] | WHERE[6] | WHEN and HOW MUCH[7] | TAILORING[8] | MODIFICATIONS[9] | HOW WELL[10] |
|---|---|---|---|---|---|---|---|---|---|
| Texas!Grow! Eat!Go (TGEG) (Van Den Berg et al., 2020) | Due to logistical issues and the expense of implementation of multiple interventions in the same school, few such interventions have been formally evaluated. The TGEG study was developed to address this gap. | The 6-month LGEG intervention included a school garden and a 32-lesson school curriculum that centered around the vegetables grown in the school gardens. During the year, students grew vegetables and participated in both fresh vegetable samples and classroom vegetable recipe demonstrations. They also took home recipe cards and Family Stories (home booklets of eight bilingual chapters on gardening, healthy cooking and family physical activity). The stories paralleled the classroom curriculum and were to be completed at home with parents. http://jmgkids.us/lgeg<br><br>Walk Across Texas program or WAT! is a school-based physical activity (PA) program, which includes multiple program components designed to establish the habit of regular PA among youth (http:// walkacrosstexas.tamu.edu)<br><br>For the TGEG study, components of the WAT! program included a kick-off event, a classroom team mileage competition, weekly lesson plans, family engagement pieces (bonus miles form), and an end-of-program celebration. Weekly English and Spanish newsletters featuring both healthy PA and eating tips were added to enhance family engagement | Texas Education Agency-approved coordinated school health (CSH) program. The gardens were built by AgriLife Extension Specialists, teachers, students, and parents. The LGEG local AgriLife Extension Educators provided the coordination and technical assistance to the teachers, Master Wellness and County Extension Educators, and volunteers on garden installation, vegetable tasting, and related vegetable recipe demonstrations.<br><br>All participating third grade teachers, school family specialists, coaches, principals, and school volunteers participated in an 8-hour hands-on training provided by the Texas A&M AgriLife Extension Service Junior Master Gardener, Nutrition and Physical Activity Specialists. | 28 schools were randomly assigned to 1 of the 4 conditions. The four conditions included (1) School Garden intervention (LGEG), (2) Physical activity intervention (WAT!), (3) both Garden and Physical activity interventions (Combined), and (4) neither a Garden nor physical activity intervention (Delayed Control). Both included face-to-face in group garden activities and classroom vegetable or physical activity events and home orientated activities to do in family. | 28 schools in Texas (USA) in school gardens, school classes and at children's home. | 6-month during the school year (implementation: 2012/2013 and 2013/2014) | No | No | Teachers provided information about fidelity of program implementation using a structured program implementation log. Teachers completed the logs by providing dates and hours related to each intervention component. Interviews were conducted with school principals regarding administrative support for the intervention implementation. AgriLife Extension Educators scored classroom implementation of key program components for each teacher using in-class and in-school observations.<br><br>Implementation fidelity in the combined schools was less complete compared to the schools with the single interventions (data |

| BRIEF NAME[1] | WHY[2] | WHAT[3] | WHO PROVIDED[4] | HOW[5] | WHERE[6] | WHEN and HOW MUCH[7] | TAILORING[8] | MODIFICATIONS[9] | HOW WELL[10] |
|---|---|---|---|---|---|---|---|---|---|
| Outdoor lessons in gardens (Rees-Punia et al., 2017) | Children with more exposure to green space accumulate more physical activity, and that gardens specifically support an environment conducive to higher intensity physical activity in children | Replacing one traditional indoor classroom lesson with an outdoor garden-based lesson. In addition to taking place in the garden, teachers were instructed to incorporate features of the garden as a teaching tool in each of the garden classes. While teachers were provided ideas for garden-based activities associated with grade-level curriculum standards, class content class content was left to teacher discretion to mimic real-world experiences and minimize disturbances to children's learning. Teachers led hands-on activities in the garden to enhance and reinforce concepts traditionally taught in the classroom. http:// extension.uga.edu/k12/sch ool-gardens/curriculum/index.cfm | Science, technology, engineering, and math teachers who were responsible for maintaining the school garden and were willing to use the garden during class time with zero to seven years years of experience teaching class in the garden. | Participating teachers agreed to teach one science or math lesson in the school garden (face-to face and in group) | 4 elementary schools in Georgia (USA) with a garden. The establishment of the garden ranged from 2008 to 2014. The square footage varied from 1,400 to 14,100. Garden amenities, such as the number of raised beds and the presence of a toolshed varied across schools. | One science or math lesson in the school garden weekly from September through November and data were collected on three occasions scheduled three to four weeks apart. Average outdoor class time ranged from 15 to 45 minutes per school. | No | Not stated | Many differences in school demographics and garden amenities |
| Outdoor educational intervention (Gustafsson et al., 2012) | To foster learning through the interactions between emotions, actions and thoughts, based on practical observation in authentic situations | The main point of the intervention was to move the education from indoors to the natural green environment. material from the natural environment was used as teaching material and the teaching was taking place in the outdoor environment. As an illustration from the mathematics lectures, branches, stones and cones were used as examples of geometrical forms. Similarly, in language classes the children made use of objects from nature and culture in their local environment to illustrate grammatical concepts. | The regular school teachers. The teachers were introduced to exercises related to the national elementary school curricula in language, mathematics, natural sciences, arts, music, drama and physical education to give the teachers insights into outdoor educational methods directly applicable in a physically active instructional setting located outside the traditional classroom environment | School work at the reference school employed traditional methods; all education was book-based and consisted of paper-and-pencil work in the indoor classroom environment. Outdoor education was used at the intervention school as a contrasting type of didactic method. At the intervention school, material from the natural environment was used as teaching material and the teaching was taking place in the outdoor environment. | The outdoor teaching of the children was mostly taking place in the surrounding forest and woods. The intervention school was located in the urban fringe of the city of Linköping (Sweden) on the countryside so has close access to forests. The reference school in the city. | Didactic sessions in the out-of-doors were held during the 6 months following the initial data collection, in total about 15 days of education. Two of these sessions consisted of whole working days while the rest mainly were held from 14.00–18.00 h in the afternoon. | No | Not stated | Not stated |

| BRIEF NAME[1] | WHY[2] | WHAT[3] | WHO PROVIDED[4] | HOW[5] | WHERE[6] | WHEN and HOW MUCH[7] | TAILORING[8] | MODIFICATIONS[9] | HOW WELL[10] |
|---|---|---|---|---|---|---|---|---|---|
| Lessons in nature (Largo-Wight et al., 2018) | Biophilia Hypothesis and Attentional Restoration Theory | Kindergarten language arts writing lesson class daily in either the indoor or outdoor classroom. The outdoor classroom was located in the school's play yard. Children sat on carpet squares and used clipboards for writing. | Two kindergarten teachers that held Bachelor's degrees in early childhood education) and English Speakers of Other languages endorsements | Teachers taught their respective kindergarten classes their regularly scheduled lesson daily in either the indoor or outdoor classroom. The two kindergarten classes rotated where they delivered the writing lessons; | Public elementary school in the southeast USA. The outdoor classroom was located in the school's play yard under a tree and shade tents defined with a circular boundary of tires, tree stumps, and potted plants. | From mid-March to mid-May, two kindergarten classes over six weeks. | No | Not stated | Not stated |
| Lessons in nature (Kuo et al., 2018) | The hypothesis is that lessons in nature have positive, immediate after effects on classroom engagement that is, when children learn outdoors, their classroom engagement after returning indoors is better than it would have been had they stayed inside the entire time. | A mini-experiment replicated 20 times, a lesson in nature vs. after a matched lesson in the classroom on the same topic, with the same teacher and students. This mini-experiment was repeated across 10 different lesson topics and weeks (one topic per week), in each of two classrooms.<br><br>For each pair of lessons, (one in nature, one in the classroom) the two teachers worked together to adapt a different theme from the Project Learning Tree (www. plt.org) environmental education lesson guide, with lessons on leaf, tree, and seed identification; organic matter decomposition; the life cycle; and pollution. | Two teachers highly experienced and state-certified in elementary education, with Masters in Education degrees and in-service training in outdoor and environmental education. These teachers had teamed together in lesson planning over a period of 5 years prior to this study, facilitating their coordination of lessons. | Compared classroom engagement after a teacher gave her students a lesson in nature vs. after the same teacher gave her students a lesson on the same topic in the classroom (e.g., leaves) in the same week, replicating this comparison across 10 different topics (one topic per week), two classrooms ("classroom a," with its own teacher, students, and room; and "classroom b," with another teacher, set of students, and room). | The indoor condition in this study comprised two typical classrooms (with windows). The outdoor condition comprised a small grassy area just outside the school. This instructional area was adjacent to a stream and woodlands, not used in the lesson. While the teacher was setting up the outdoor lesson, students occasionally visited the stream bank briefly. To reach outdoor site students walked about 200 meters. | 40-min lesson in nature + walking to the outdoor site. The lesson delivered in nature was roughly 30 min long whereas the matched indoor lesson was 40 min long. The observation period for both conditions was 20 min long over 10 different weeks in the semester from September-November, under a range of weather conditions. | No | One occasion, a planned lesson was not given as scheduled; that lesson was made up in April instead. Analyses with and without the makeup lesson and its paired classroom lesson show the same effects of lessons in nature on subsequent classroom engagement | Not stated |

| BRIEF NAME[1] | WHY[2] | WHAT[3] | WHO PROVIDED[4] | HOW[5] | WHERE[6] | WHEN and HOW MUCH[7] | TAILORING[8] | MODIFICATIONS[9] | HOW WELL[10] |
|---|---|---|---|---|---|---|---|---|---|
| Lessons in nature<br><br>(Taylor and Butts-Wilmsmeyer, 2020) | Attention Restoration Theory | All classes in the study were already using their school's green outdoor classroom and other greenspaces for curriculum at least once per week; the study intervention prescribed the frequency and duration of those sessions. There were two conditions of prescribed use –'Low Frequency' (once per week) or 'High Frequency' (daily). Pavement and fences were removed and replaced with softer surfaces (grass, sand, or mulch), and young trees were planted. Play elements were also installed and could include: a series of stumps for climbing and jumping, a low wooden platform for a stage, tires and sand embedded into a hillside for climbing, sandboxes, garden boxes, and semi-circular seating areas. All schools also had, in addition to the outdoor classroom, paved areas for play surrounded by natural elements. | Educators who had taught full-day Kindergarten for at least two years. All had received training in inquiry-based learning, which centers on children's questions and first-hand exploration of a topic and currently practiced inquiry-based learning methods in both the indoor and the green outdoor classroom. Educators were trained by the first author to implement the intervention. All educators were fully trained in and had been teaching self-regulation techniques for several years before the start of the study. | Classes in the Low Frequency condition (both winter/spring and fall) committed to no more than one 60-minute greenspace curriculum session per week. Classes in the High Frequency condition in the winter/spring study were prescribed to a minimum of 30 minutes of greenspace curriculum daily – then increased to increase to a minimum of 60 minutes daily | Outdoor classroom was defined as a portion of the schoolyard, adjacent to the building, that had previously been paved and fenced. Nearby naturalized areas were typically a dense stand of trees including understory and paths throughout, and a few also had an open field, that was owned by the school or by the community. In the winter/ spring sample, one school had access to an adjacent naturalized area, and one school had community parks several blocks away. In the fall study, all schools had access to a naturalized area, but for some schools, those areas were several blocks away. | In the winter/spring study the High Frequency classes engaged in greenspace curriculum on average from 3 to 5h per week, while low frequency 25.4 min per week. In fall, the High Frequency classes engaged in greenspace curriculum on average 4–7 h per week, while low frequency on average 40 min per week | No | Both Low and High Frequency classes were spending more total minutes outdoors each day (e.g., multiple recesses) than anticipated, thus potentially diluting any impact. In the fall study, the prescribed minutes of greenspace curriculum for High Frequency classes was increased to a minimum of 60 minutes daily. | The quality of the nature experienced ranged from young trees and mature trees in the near and distant view, to paths through deep woods and open lawn for gathering or focused play |
| Urban forests / Forest Therapy<br><br>(Bang et al., 2018) | Natural environment may be the best place to regain our attention and focus, and these benefits have also been shown to occur in children. | Health promotion program using urban forests consisted of lectures on physical and psychosocial health and forest experience activities. A written program manual was developed for the intervention providers to ensure adherence to the intervention protocol and consistency throughout the program | The program was conducted by the research team, all of whom were registered nurses whose majors were either child health or psychiatric nursing. One of them was a certified forest therapist, and one had completed the approved training course for forest therapy. A total of seven nursing student mentors were involved in the activities | 10-session health promotion program using urban forests consisted of lectures on physical and psychosocial health and forest experience activities. Each session allocated 30 min for the lecture and 60 min for the forest activities in the urban forests, which were 10 min away from each community center for children. | Urban forest which was 10 min away from each community center for children. The control groups attended only the routine programs (e.g., supplementary learning such as math or English, reading, art) at their community center for children | From June to August 2017 over 10 weeks | No | Not stated | Not stated |

| BRIEF NAME[1] | WHY[2] | WHAT[3] | WHO PROVIDED[4] | HOW[5] | WHERE[6] | WHEN and HOW MUCH[7] | TAILORING[8] | MODIFICATIONS[9] | HOW WELL[10] |
|---|---|---|---|---|---|---|---|---|---|
| Wilderness Schooling (WS) (Quibell et al., 2017) | There is a gap in outdoor learning research for more rigorous experimental investigation of the impact of outdoor learning on academic attainment. | Wilderness Schooling is a manualised programme of lesson plans and activity resources to guide a programme of curriculum delivery lasting six days. During intervention days, children took part in several different tasks outdoors. Days were structured to each have an individual curriculum flavour; two days were science days, two English and two math | Each Wilderness Schooling programme was delivered by two Wilderness Practitioners qualified primary or secondary school teacher and the other an assistant with interest and experience in working outdoors with children. Two days of training in the WS delivery materials was given | Matched-groups design was implemented, whereby one group received the WS intervention and a comparison group remained in school and received conventional classroom-based learning. Teachers were asked to divide their class of balance-gender 30 children as evenly as possible into two groups. WS intervention was structured as introduction to the day with circle-time activities to promote group bonding, identification of a curriculum question, leading straight on to tasks invariably conducted outdoors—searching, gathering data, measuring or collecting experiences and sense-impressions in note form, this process might continue beyond lunch, after which the children are taken outdoors for creative expressive time that might include a presentation of group learning from the curriculum task. Campfires, story- telling and art activities all feature | National Trust site | One day a week for six consecutive weeks (36 hours in all for each child) | No | Not stated | Not stated |

| BRIEF NAME[1] | WHY[2] | WHAT[3] | WHO PROVIDED[4] | HOW[5] | WHERE[6] | WHEN and HOW MUCH[7] | TAILORING[8] | MODIFICATIONS[9] | HOW WELL[10] |
|---|---|---|---|---|---|---|---|---|---|
| Green breaks (Amicone et al., 2018) | Attention Restoration and the Stress Reduction Theory | Play in the natural environment (garden) or in the built (courtyard) environment of the school. A brief description of the study was provided through the informed consent sheet. In the first experiment, children were asked to play a competitive team-game, and in the second to free-play. | Researchers (funded by Sapienza University Grants) | Study 1: Children were enrolled in usual school activities starting at 8:30 a.m. then, they completed the three paper-and-pencil measures of attention immediately pre and post their 30 minutes break time, in either: competitive play in the natural environment (garden) or in the built (courtyard) environment of their school.<br><br>Study 2: From 8:30 a.m. to 1:00 p.m., children were enrolled in usual school activities. From 1:00 p.m. to 2:00 p.m., children performed the attention test. children had their recess time from 2:00 p.m. to 2:30 p.m. (free play in a natural environment vs. built environment). From 2:30 p.m. to 4:30 p.m post-test was administered | Two Italian primary schools. Study 1: school garden (1,303 m2), the built one is the courtyard in front of the school entrance (139 m )<br><br>Study 2: The two areas used for the experiment have almost equal dimensions (around 460 $m^2$) and are close to each other (natural elements are visible from the built area and vice versa) | Study 1: March–April 2014 on two different weekdays.<br><br>Study 2: end of May 2016 | No | For study 2: the experiment was conducted in the afternoon school time when children may need more restoration | Not stated |

| BRIEF NAME[1] | WHY[2] | WHAT[3] | WHO PROVIDED[4] | HOW[5] | WHERE[6] | WHEN and HOW MUCH[7] | TAILORING[8] | MODIFICATIONS[9] | HOW WELL[10] |
|---|---|---|---|---|---|---|---|---|---|
| School playing environment (Wood et al., 2014) | Performing physical in a natural environment (''Green Exercise'') can encourage unstructured play and may therefore play a role in facilitating unstructured physical activity during playtime | Over two consecutive weeks participants were allocated to either play on the school field or the playground during morning and lunch playtime.<br><br>The selected school has extensive outdoor areas, including three playgrounds, a garden area, an environmental area, an outdoor classroom and large grassed field. | Researchers (Funded Heart Research UK) | Participants were first grouped according to whom they would normally play with during playtime. The order in which these groups played in the two areas was then randomised and counterbalanced to eliminate any order effects, with approximately half of the group playing in each area at any one time. Participants were instructed to play as normal and were free to engage in their chosen activities. Participants could not see the playground from the field; however the boundary of the field was visible from some areas of the playground | In one school in the urban area of UK. The school field was surrounded by trees and bushes, whilst the playground consisted of concrete areas surrounded by school buildings | Two consecutive weeks during morning and lunch playtime. Morning playtime lasted for 15 minutes, whilst lunch playtime lasted for one hour including the time taken to eat lunch (approximately 30 minutes). | No | Not stated | Not stated |
| Nature-based Playground<br><br>(Barton et al., 2015) | Accessing green space and engaging in outdoor play during the school day is a key component to the health, well-being, and development of children | Two playtime interventions were then introduced into each of the schools during lunch playtime: A playground sports equipment intervention was performed on the playground and consisted of small pieces of equipment such as skipping ropes, bats and balls, and Frisbees. Orienteering intervention was carried out on the school field and green areas surrounding the school buildings. Children were provided with a map of the school grounds and a course with markers to follow and the course was altered on each day of the intervention | Researchers (Funded by Heart Research UK) | The interventions were available for the whole 55-minute duration of lunch playtime on five consecutive days. A playground sports equipment intervention was implemented followed by an orienteering intervention (each intervention with one week gap in between). The order was requested by the school due to circumstances out of our control. | In the school playground and field. The dimensions of the green and playground areas were comparable, and all children participated fully in both interventions | In two primary schools in socially deprived urban and rural areas in England. During lunch playtime for one week between November and December 2009. | Not stated | Not stated | Not stated |

| BRIEF NAME[1] | WHY[2] | WHAT[3] | WHO PROVIDED[4] | HOW[5] | WHERE[6] | WHEN and HOW MUCH[7] | TAILORING[8] | MODIFICATIONS[9] | HOW WELL[10] |
|---|---|---|---|---|---|---|---|---|---|
| Playground greening project<br><br>(Raney et al., 2019) | No study has examined a stepwise impact of school playground design changes or deter-mined whether PA rates persist over time for individual students. Studies comparing sex-specific and age-group physical activity levels in green schoolyards are also lacking | Aprox. 21,000 square feet of asphalt distributed in four playground zones were replaced by green space.<br><br>At baseline, the following zones were present at both control and intervention school:<br>kickball, basketball, volleyball, four-square, dodgeball, tetherball, handball, open field, and non-designated space near buildings. | Researchers (No funding reported) | Changes included (1) introduction of trees, mulch, and boulders in two zones; (2) replacement of one asphalt field with grass and trees;(3) replacement of another asphalt field with an outdoor class-room (decomposed granite floor, mulch and plant border, and log seating) | In a playground of one title I elementary school in Los Angeles, California. | In summer 2016 | No | Not stated | Not stated |
| Schoolyard Greening<br>(van Dijk-Wesselius et al., 2018) | Biophilia hypothesis, stress recovery Theory, Attention Restoration Theory | The greening of the five schoolyards between baseline and first follow-up was a tailored process. Guidelines for greening schoolyards developed by Fonds1818 Dutch foundation, including grassy hills, bushes, tree, tunnels made of tree branches, loose tree branches and garden-like parts. A pre-plan design was approved by the foundation, then the greening was carried out in a participatory process with input from parents, teachers, children and designers. | Fonds1818 Dutch foundation which has subsidized greening of 187 schoolyards in the Western part of the Netherlands | Paved schoolyards were mostly covered with tiles and contained some play equipment made of non-natural materials, like swings or climbing frames. When vegetation was present, this served only as a fence or decoration. All intervention schools greened areas of their schoolyard and also kept some areas paved. The green areas covert mostly features as grassy hills, bushes, tree, tunnels made of tree branches, loose tree branches and garden-like parts. | 5 schoolyards from elementary schools in moderate-to-high-urbanized areas in The Netherlands | 2015 | No | Not stated | Although, all intervention schools had plans to substantially green their schoolyards, the actual greening was modest in some cases and all greened schoolyards still contained some paved areas. |
| Green Walls in Classrooms in Haarlemmermeer<br>(van den Berg et al., 2017) | Stress Recovery Theory, Attention Restoration Theory | Green walls were of the type "Wall so green". A metal frame with layers of felt with fertile soil for the plants. The unit was stocked with eight types of green plants, including Spathiphyllum, Philodendron, Dracaena | Researchers (funded by Greenport Aalsmeer) | Group-wise intervention face-to-face: in each classroom, a single wall unit of 1.25 m wide and 2 m high was placed in the back of the room against the rear wall or in one of the corners against a sidewall. | 8 classrooms (4 control) from two elementary schools in The Netherlands with different windows' views (i.e., parking lot, grass filed, backside of residential houses) | October 2014 the green wall was installed (2 and 4 months after placement follow-ups) | No | Not stated | Not stated |

| BRIEF NAME[1] | WHY[2] | WHAT[3] | WHO PROVIDED[4] | HOW[5] | WHERE[6] | WHEN and HOW MUCH[7] | TAILORING[8] | MODIFICATIONS[9] | HOW WELL[10] |
|---|---|---|---|---|---|---|---|---|---|
| Greenery on the Classroom<br><br>(Bernardo et al., 2021) | Attentional Restoration Theory | Artificial green wall, 150 cm wide and 250 cm in height, and lettuce in a pot | Teachers and Researchers FCT (Portuguese Foundation for Science and Technology) | 1st assessment, placement of an artificial green wall, 2nd assessment after one moth, an activity in which the children planted one lettuce in a pot and was responsible for maintaining its own lettuce, namely watering it and monitoring its growth, 3rd assessment. | Study 1: 4 classrooms from a middle-class public primary school in Lisboa;<br><br>Study 2: 2 classrooms from 2 schools located in: low and middle-class (same study 1) areas of Lisboa | October/Dec 2019 – assessments one and two months after interventions | No | Not stated | Not stated |
| Safe Routes to School (SRTS) programs (Stewart et al., 2014) | To offer the first evaluation of the state-level SRTS program. | Infrastructure or non-infrastructure activities. **Infrastructure** included sidewalk or crosswalk construction, installation of permanent signages, construction of a shared use path, bicycle lane installation, and construction of pedestrian overpasses or bridges.<br>**Non -infrastructure** activities included media campaigns or promotions to the general public; increased police patrol with emphasis on pedestrian and bicycle safety; walk or ride to school day events; walking school buses; mileage clubs or pedometer programs that reward children for accumulating active travel; bicycle rodeos, which are special events to teach bike skills; additional crossing guard personnel; bicycle equipment such as helmets or locks; | US Department of transportation<br><br>State-funded SRTS project | State-wise and individual strategies (i.e., bicycle equipment)<br>Higher percentage of combined activities, a lower percentage of infrastructure, and no non-infrastructure projects. Three mostly common infrastructure projects: sidewalks, crosswalk, signage. | 48 SRTS projects covering 53 schools across Florida, Mississippi, Washington, and Wisconsin | From the beginning of the federal SRTS program in 2005 through April 15, 2011, in five of the participating states. | No | Not stated | Not stated |

| BRIEF NAME[1] | WHY[2] | WHAT[3] | WHO PROVIDED[4] | HOW[5] | WHERE[6] | WHEN and HOW MUCH[7] | TAILORING[8] | MODIFICATIONS[9] | HOW WELL[10] |
|---|---|---|---|---|---|---|---|---|---|
| Safe Routes to School (SRTS) program | More research is needed on the impacts of the SRTS program using stronger research designs<br><br>While previous research has suggested positive program impacts, there have been no large-scale studies with strong research designs | Interventions are broadly classified into 4 categories, also known as the 4E's: engineering, education, encouragement, and enforcement. Engineering interventions are usually infrastructure improvements. Education programs aim to improve students' active commuting skills and awareness, as well as increase the safety of walking and biking activities. Similarly, encouragement interventions seek to raise the awareness of active commuting benefits among both students and their parents. Enforcement includes intervention measures such as the funding of street crossing guards, the placement of speed feedback trailers near schools, and increased police presence to enforce speed limits in school zones | US Department of transportation Safe Routes to School Programs | | | | | | |
| (McDonald et al., 2013) | | Side-walk construction, crosswalks, and traffic signal improvements. https://www.oregonsaferoutes.org/ | | Most school district SRTS interventions have been in the categories of education, encouragement, and engineering. Two middle schools, five elementary, and one K-8 school have education and encouragement programs. One middle school took part in the Boltage program, which encourages biking offering prizes based on participation. Infrastructure changes were made at several schools, ranging from improved bike parking at three schools to | Nine 4J elementary schools and four middle schools in Eugene, Oregon (USA) | 2007-2011 The oldest intervention began in fall 2007 and have generally been delivered consistently during the study period. The bike parking improvements were made early in the program; the crosswalk and sidewalk improvements were | No | Not stated | Eugene program has evolved slowly. Most early efforts focused on education and encouragement; infrastructure improvements – with their long planning timelines – were implemented later |

| | | | | | | | | |
|---|---|---|---|---|---|---|---|---|
| | | | | crosswalk and sidewalk improvements at five schools. | | implemented during summer 2011 and, therefore, were not available during much of the study period. | | |
| (McDonald et al., 2014) | | Small rewards such as pencils and stickers, or using organized to encourage children to walk. | | In this sample, many schools reported sidewalk and crosswalk improvements but relatively few investments in bicycle lanes or off-street paths. | 801 (n=423 controls) schools in DC, Florida, Oregon, and Texas | 2007–2012 | No | Not stated | Not stated |
| T-COPPE Texas Childhood Obesity Prevention Policy Evaluation<br><br>(Hoelscher et al., 2016) | Schools with SRTS infrastructure funding would have a significantly greater percentage of children engaged in active travel compared with schools with SRTS noninfrastructure funding and schools with no SRTS funding, and (b) schools with SRTS noninfrastructure funding would have a significantly greater percentage of children engaged in active travel compared with students in schools with no SRTS funding | The majority of projects (78%, n = 194) were awarded noninfrastructure projects, which included (a) local program and plan development or (b) local implementation. Of the noninfrastructure projects, 170 (68% of the total awards) were funded for development of an SRTS plan, while 24 were funded for local implementation, which included educational, encouragement, enforcement, and evaluation activities. The remainder of the projects (22%, n = 56) were awarded as infrastructure grants, which included engineering project(s) to allow active travel, such as sidewalks, crosswalks, and so forth. http://go.uth.edu/TCOPPE | Texas Department of Transportation | Projects in communities through individual or district wide grants on a cost-reimbursement basis<br><br>Noninfrastructure schools had to submit an SRTS plan although implementation of the plan was not required.<br><br>Infrastructure schools were required to have an SRTS plan in place prior to any structural changes and had several years to complete the planned environmental changes. | 78 elementary schools (n=34 control) in Texas State (US) | 2009 - 2012 | No | Not stated | Measures of implementation were not included. But, qualitative indicated that infrastructure projects were not always completed by the time of the follow-up survey |

| BRIEF NAME[1] | WHY[2] | WHAT[3] | WHO PROVIDED[4] | HOW[5] | WHERE[6] | WHEN and HOW MUCH[7] | TAILORING[8] | MODIFICATIONS[9] | HOW WELL[10] |
|---|---|---|---|---|---|---|---|---|---|
| School Travel Plan (STP) | To determine the effectiveness of the STP program in changing school travel modes in children<br><br>How has participation in active commuting changed over time when compared to before STP implementation? 2) How does the effect of the STP on active commuting differ by school year, socioeconomic status, and school size? | STP program is the collaborative approach during planning; a travel planner (assigned to each school), school staff, council, local community, and other stakeholders work together to develop tailor-made initiatives that are appropriate for the culture of each school and the wider community. The program included a combination of infrastructural and educational measures across schools: engineering (crossings, cycling facilities, speed-reduction devices, road markings, and signage); Road safety education; Enforcement activities; Public education; Rewards and awards; Policy (No on-site parking space for student drop-offs will be provided.) | New Zealand Transport Agency and facilitated by partnerships between the Auckland Regional Transport Authority (ARTA) and local councils | The STP planning process involved four phases: (1) set-up, (2) data collection, (3) planning, and (4) implementation and monitoring. During the set-up phase, the school, local council, and ARTA representatives decided on an overall timeframe, key aims, and an evaluation framework. Transport and safety issues were identified by gathering information about the school and the community, collating and analyzing data from the baseline travel survey, consulting with the community, and providing feedback to an STP working group. Once the pertinent issues were identified, the travel coordinator facilitated the development of an action plan that used a combination of engineering, education, enforcement, encouragement, and policy strategies. In the last phase of the process, the travel coordinator assisted with the implementation of all aspects of the action plan and informed the school community of the impact of the implemented strategies after follow-up data collection. | Elementary schools across the Auckland region - agglomeration of urban cities and towns including Auckland, North Shore, Waitakere, and Manukau cities, and the peri-urban areas of Papakura, Rodney, and Franklin Districts (New Zealand) | | | | While all schools followed the standard planning process, each school implemented the STP at different times. |
| (Hinckson and Badland, 2011) | | | | | 33 elementary schools | Between 2004 and 2008 | No | Not stated | Not stated |
| (Hinckson et al., 2011) | | | | | 56 schools' elementary schools | Between 2004 and 2006 (STP implementation) | No | Not stated | Not stated |

| BRIEF NAME[1] | WHY[2] | WHAT[3] | WHO PROVIDED[4] | HOW[5] | WHERE[6] | WHEN and HOW MUCH[7] | TAILORING[8] | MODIFICATIONS[9] | HOW WELL[10] |
|---|---|---|---|---|---|---|---|---|---|
| Safe Routes to School (SR2S) (Ragland et al., 2014) (only Mobility study) | To develop analyses that were location-specific, that is, to look at safety and mobility near specific SR2S infrastructure improvement | The California program provides funding to municipalities for engineering modifications such as sidewalks, crosswalk placement and painting, traffic lights, and speed humps near schools. The municipality is required to provide a minimum of 10% in local matching funds. A funded project at a school site can list zero, one, or multiple countermeasures. For example, an SRTS project could fund the construction of sidewalks, curb ramps, and radar speed feedback signs for a school. One countermeasure could affect multiple schools. For example, a project could fund the construction of a sidewalk expansion that would affect two schools that are close to each other. | California State and municipalities | Countermeasure Installed: Install sidewalk (to avoid walking along roadway), Install traffic signal, install dynamic advance intersection warning system, install flashing beacons as advance warning, replace existing “Walk–Don’t Walk” signals with pedestrian countdown signal heads, install speed humps, install changeable speed warning signs for individual drivers, Improve superelevation (for drainage) | Schools areas that were affected by SRTS projects in 47 schools across (nine schools from southern California were included in the mobility study): 250 ft of the countermeasure versus households farther than 250 ft but less than a quarter mile from the school | 10 years | No | Not stated | Not stated |
| School travel plan (STP) (Buliung et al., 2011) | There is growing interest in understanding if and how active school transportation enhances children’s physical activity. Evidence for school active travel program efficacy is rather mixed. | School travel plans were developed at all schools; key elements of the plans and implementation frequency. Interventions were classified using 4 categories: (1) education, (2) activities and events, (3) capital improvement projects, and (4) enforcement | Green Communities Canada granted by the Public Health Agency of Canada to pilot an STP framework across Canada. Led by the trained STP facilitator, participating schools, assisted by community stakeholders, were guided through a reflective and iterative 5-stage STP process | Infrastructure improvements generally involved installation or repainting of crosswalk lines, removal of physical barriers preventing access to sidewalks and walkways (shrubbery and snow), installation of 4-way stops and streetlights, repair of damaged walkways, and increased school zone signage to make changes in posted rates of speed more visible to drivers. Most frequently implemented was the painting of crosswalk lines at intersections. This was followed by the introduction or improvement of signage demarcating school and parking zones. More costly interventions such as sidewalk installation or repair were practiced less frequently. | 12 schools (3 per province) across 4 provinces (Alberta, Nova Scotia, Ontario, British Columbia) | November 2007 and November 2009 | Not stated | Not stated | Not stated |

| BRIEF NAME[1] | WHY[2] | WHAT[3] | WHO PROVIDED[4] | HOW[5] | WHERE[6] | WHEN and HOW MUCH[7] | TAILORING[8] | MODIFICATIONS[9] | HOW WELL[10] |
|---|---|---|---|---|---|---|---|---|---|
| Changing Road Infrastructure (Smith et al., 2020) | Knowledge gaps exist in terms of how such small-to-medium scale, school-focused infrastructural changes might impact children's active school travel and variables along the pathway to behavior change in New Zealand. The intervention consisted of three stages: investigation, detailed design and formal community consultation, and construction. The intervention was also designed to work in parallel with existing road safety education and active school travel encouragement Initiatives | Road safety messages in school newsletters, promotions to park further from the school and walk the remaining distance, promotions to encourage parents to drive slowly and park safely if near school entrances, and student leadership groups. Some schools also patrolled school crossings and provided bike and scooter parking. Treatments had to fall below a cost threshold, with larger projects falling outside the budget scope of the program (NZ$700,000). | Auckland Transport | Neighborhood street infrastructural changes for improved safety. Infrastructural elements: Relocation of crossing to improve visibility of pedestrians by oncoming and turning motorists, Pram crossings and tactile paving added, Formalised bus stop through painting road markings, Pedestrian refuge island installed including pram crossings and tactile paving, Pedestrian refuge added to existing median barrier, path upgrade, and removal of corner barrier to improving safe and, Pedestrian refuge island, pram crossings, and tactile paving added, Roundabout installation with pedestrian refuges on each approach, pram crossings and tactile paving, Installation of four-speed humps, with cycle cut-throughs | Across local community, with schools as a focal point (surrounding school roads/streets) | Infrastructural works were delivered from November 2016 to May 2017 | No | Not stated | Not stated |
| "Tryg og Sikker Skolecykling" (Safe and secure cycling to school) (Ostergaard et al., 2015) | walking and cycling to school are two different behaviours with specific determinants | Planned infrastructural changes near schools (e.g., road surface and traffic regulation) and school-motivation for promoting commuter cycling | Local authorities and The Danish Cyclists Federation | Hard interventions included changes near the school in e.g., road surface, signposting and traffic regulation such as one-way streets and regulation of car commuting drop off zones. Soft interventions included a variety of different school cycling incentives | structural changes near the school | April and May 2010 with follow-up one year after intervention | No | the timing of baseline measurements became incompatible with the construction work we had difficulties passing on the scientific importance that control schools were not offered any cycling interventions. | intensity of interventions was determined as a score from 1-5 by the provider: Intensity of "hard" interventions ranged 0-5 |

| BRIEF NAME[1] | WHY[2] | WHAT[3] | WHO PROVIDED[4] | HOW[5] | WHERE[6] | WHEN and HOW MUCH[7] | TAILORING[8] | MODIFICATIONS[9] | HOW WELL[10] |
|---|---|---|---|---|---|---|---|---|---|
| School travel plan (STP) | Evidence provides little indication of which types of children, families, and/or schools would benefit most from STP. More specifically, none of the STP studies explicitly examined a combination of child, family, and school-level characteristics that may indicate greater travel mode change from driving to active travel post-intervention | School travel planning is a multi-disciplinary, multi-sectoral, school-specific intervention that engages key stakeholders in the survey and evaluation of school travel issues. Subsequently, stakeholders develop and implement an action plan with the objective of increasing active school travel at that specific school. Typically: educational strategies (e.g., hosting educational workshops to promote the awareness and benefits of active travel); activities and events (e.g., organizing active travel supervision via walking school bus schemes); capital improvement projects (e.g., installing a sidewalk or bike rack); and enforcement initiatives (e.g., increased police presence or crossing guards). | Green Communities Canada (ONG) at the national level followed by provincial or territorial organizations, local school facilitators, and school stakeholder committees (e.g., STP facilitator, public health, police officials, municipal planners and traffic engineers, school boards, parents, children, school administrators and teachers | Face-to-face, classroom and student-level activities related to (a) infrastructure modifications/additions; (b) safety education; (c) special walking events; (d) walking buddies/walking schoolbus formation; (e) active travel newsletter dissemination and (f) identification of best routes to school | | January 2010 and March 2012 | | | Uncontrollable factors also influenced implementation. The teacher's strike in British Columbia meant that all "non-essential" duties could not be completed in the academic year. As a result, the STP intervention was not implemented in 12 schools in this province. Due to the limited timeframe, half of the schools also failed to submit complete data and could not be used for analysis |
| (Mammen et al., 2014a) | The STP process may have a differential impact on mode share during the trip to and from school. To identify components of the action plans were indicative of active travel change post-intervention.. | | | | School-level and their surroundings in 103 public elementary schools across Canada | | No | Not sated | |
| (Mammen et al., 2014b) | | An honorarium of $1000 at completion of baseline and follow-up surveys was received by the schools which could be used to support active travel initiatives at the school level | | | School-level and their surroundings in 106 public elementary schools across Canada | | No | Degree of STP implementation was not assessed | . |

[1]Provide the name or a phrase that describes the intervention.

[2]Describe any rationale, theory, or goal of the elements essential to the intervention.

[3] Materials: Describe any physical or informational materials used in the intervention, including those provided to participants or used in intervention delivery or in training of intervention providers. Provide information on where the materials can be accessed (e.g., online appendix, URL). Procedures: Describe each of the procedures, activities, and/or processes used in the intervention, including any enabling or support activities.

[4] For each category of intervention provider (e.g., psychologist, nursing assistant), describe their expertise, background and any specific training given.
[5] Describe the modes of delivery (e.g., face-to-face or by some other mechanism, such as internet or telephone) of the intervention and whether it was provided individually or in a group.
[6] Describe the type(s) of location(s) where the intervention occurred, including any necessary infrastructure or relevant features.
[7]Describe the number of times the intervention was delivered and over what period of time including the number of sessions, their schedule, and their duration, intensity or dose.
[8] If the intervention was planned to be personalised, titrated or adapted, then describe what, why, when, and how.
[9] If the intervention was modified during the course of the study, describe the changes (what, why, when, and how).
[10] Planned: If intervention adherence or fidelity was assessed, describe how and by whom, and if any strategies were used to maintain or improve fidelity, describe them. Actual: If intervention adherence or fidelity was assessed, describe the extent to which the intervention was delivered as planned.

BMI = Body mass index; HVAC = Heating, Ventilating, and Air Conditioning; $CO_2$ – carbon dioxide; $NO_2$ - Nitrogen dioxide; $O_3$ -Ozone; $PM_{2.5}$ - particulate matter ≤ 2.5 micrometers; $PM_{10}$ - particulate matter ≤ 10 micrometers; MVPA = Moderate to Vigorous Physical Activity

## REFERENCES


Amicone, G., Petruccelli, I., De Dominicis, S., Gherardini, A., Costantino, V., Perucchini, P., Bonaiuto, M., 2018. Green Breaks: The Restorative Effect of the School Environment's Green Areas on Children's Cognitive Performance. Front. Psychol. 9. https://doi.org/10.3389/fpsyg.2018.01579

Bakó-Biró, Z., Clements-Croome, D.J., Kochhar, N., Awbi, H.B., Williams, M.J., 2012. Ventilation rates in schools and pupils' performance. Build. Environ. 48, 215–223. https://doi.org/10.1016/j.buildenv.2011.08.018

Bang, K.-S., Kim, S., Song, M.K., Kang, K.I., Jeong, Y., 2018. The Effects of a Health Promotion Program Using Urban Forests and Nursing Student Mentors on the Perceived and Psychological Health of Elementary School Children in Vulnerable Populations. Int. J. Environ. Res. Public Health 15. https://doi.org/10.3390/ijerph15091977

Barton, J., Sandercock, G., Pretty, J., Wood, C., 2015. The effect of playground- and nature-based playtime interventions on physical activity and self-esteem in UK school children. Int. J. Environ. Health Res. 25, 196–206. https://doi.org/10.1080/09603123.2014.915020

Bernardo, F., Loupa-Ramos, I., Matos Silva, C., Manso, M., 2021. The Restorative Effect of the Presence of Greenery on the Classroom in Children's Cognitive Performance. Sustain. . https://doi.org/10.3390/su13063488

Block, K., Gibbs, L., Staiger, P.K., Gold, L., Johnson, B., Macfarlane, S., Long, C., Townsend, M., 2012. Growing Community: The Impact of the Stephanie Alexander Kitchen Garden Program on the Social and Learning Environment in Primary Schools. Heal. Educ. Behav. 39, 419–432. https://doi.org/10.1177/1090198111422937

Buliung, R., Faulkner, G., Beesley, T., Kennedy, J., 2011. School Travel Planning : Mobilizing School and Community Resources to Encourage Active School Transportation. J. Sch. Health 81, 704–712.

Gustafsson, P.E., Szczepanski, A., Nelson, N., Gustafsson, P.A., 2012. Effects of an outdoor education intervention on the mental health of schoolchildren. J. Adventure Educ. Outdoor Learn. 12, 63–79. https://doi.org/10.1080/14729679.2010.532994

Hinckson, E.A., Badland, H.M., 2011. School travel plans: preliminary evidence for changing school-related travel patterns in elementary school children. Am. J. Health Promot. 25, 368–371. https://doi.org/10.4278/ajhp.090706-ARB-217

Hinckson, E.A., Garrett, N., Duncan, S., 2011. Active commuting to school in New Zealand Children (2004-2008): A quantitative analysis. Prev. Med. (Baltim). 52, 332–336. https://doi.org/10.1016/j.ypmed.2011.02.010

Hoelscher, D., Ory, M., Dowdy, D., Miao, J.G., Atteberry, H., Nichols, D., Evans, A., Menendez, T., Lee, C., Wang, S.J., 2016. Effects of Funding Allocation for Safe Routes to School Programs on Active Commuting to School and Related

Behavioral, Knowledge, and Psychosocial Outcomes: Results From the Texas Childhood Obesity Prevention Policy Evaluation (T-COPPE) Study. Environ. Behav. 48, 210–229. https://doi.org/10.1177/0013916515613541

Kuo, M., Browning, M.H.E.M., Penner, M.L., 2018. Do lessons in nature boost subsequent classroom engagement? Refueling students in flight. Front. Psychol. 8, 1–15. https://doi.org/10.3389/fpsyg.2017.02253

Largo-Wight, E., Guardino, C., Wludyka, P.S., Hall, K.W., Wight, J.T., Merten, J.W., 2018. Nature contact at school: The impact of an outdoor classroom on children's well-being. Int. J. Environ. Health Res. 28, 653–666. https://doi.org/10.1080/09603123.2018.1502415

MacNeill, M., Dobbin, N., St-Jean, M., Wallace, L., Marro, L., Shin, T., You, H., Kulka, R., Allen, R.W., Wheeler, A.J., 2016. Can changing the timing of outdoor air intake reduce indoor concentrations of traffic-related pollutants in schools? Indoor Air 26, 687–701. https://doi.org/10.1111/ina.12252

Mammen, G., Stone, M.R., Buliung, R., Faulkner, G., 2014a. School travel planning in Canada: Identifying child, family, and school-level characteristics associated with travel mode shift from driving to active school travel. J. Transp. Heal. 1, 288–294. https://doi.org/10.1016/j.jth.2014.09.004

Mammen, G., Stone, M.R., Faulkner, G., Ramanathan, S., Buliung, R., O'Brien, C., Kennedy, J., 2014b. Active school travel: an evaluation of the Canadian school travel planning intervention. Prev. Med. (Baltim). 60, 55–59. https://doi.org/10.1016/j.ypmed.2013.12.008

McDonald, N., Yang, Y., Abbott, S.M., Bullock, A.N., 2013. Impact of the Safe Routes to School program on walking and biking: Eugene, Oregon study. Transp. Policy 29, pp 243-248.

McDonald, N.C., Steiner, R.L., Lee, C., Smith, T.R., Zhu, X., Yang, Y., 2014. Impact of the safe routes to school program on walking and bicycling. J. Am. Plan. Assoc. 80, 153–167. https://doi.org/10.1080/01944363.2014.956654

Oh, H.J., Nam, I.S., Yun, H., Kim, J., Yang, J., Sohn, J.R., 2014. Characterization of indoor air quality and efficiency of air purifier in childcare centers, Korea. Build. Environ. 82, 203–214. https://doi.org/10.1016/j.buildenv.2014.08.019

Ostergaard, L., Stockel, J.T., Andersen, L.B., 2015. Effectiveness and implementation of interventions to increase commuter cycling to school: a quasi-experimental study. BMC Public Health 15. https://doi.org/10.1186/s12889-015-2536-1

Pacitto, A., Amato, F., Moreno, T., Pandolfi, M., Fonseca, A., Mazaheri, M., Stabile, L., Buonanno, G., Querol, X., 2020. Effect of ventilation strategies and air purifiers on the children's exposure to airborne particles and gaseous pollutants in school gyms. Sci. Total Environ. 712, 135673. https://doi.org/10.1016/j.scitotenv.2019.135673

Petersen, S., Jensen, K.L., Pedersen, A.L.S., Rasmussen, H.S., 2016. The effect of increased classroom ventilation rate indicated by reduced CO2 concentration on the performance of schoolwork by children. Indoor Air 26, 366–379. https://doi.org/10.1111/ina.12210

Quibell, T., Charlton, J., Law, J., 2017. Wilderness Schooling: A controlled trial of the impact of an outdoor education programme on attainment outcomes in primary school pupils. Br. Educ. Res. J. 43, 572–587. https://doi.org/10.1002/berj.3273

Ragland, D.R., Pande, S., Bigham, J., Cooper, J.F., 2014. Examining long-term impact of California safe routes to school program: Ten years later. Transp. Res. Rec. 2464, 86–92. https://doi.org/10.3141/2464-11

Raney, M.A., Hendry, C.F., Yee, S.A., 2019. Physical Activity and Social Behaviors of Urban Children in Green Playgrounds. Am. J. Prev. Med. 56, 522–529. https://doi.org/10.1016/j.amepre.2018.11.004

Rees-Punia, E., Holloway, A., Knauft, D., Schmidt, M.D., 2017. Effects of school gardening lessons on elementary school children's physical activity and sedentary time. J. Phys. Act. Health 14, 959–964. https://doi.org/10.1123/jpah.2016-0725

Rosbach, J., Krop, E., Vonk, M., van Ginkel, J., Meliefste, C., de Wind, S., Gehring, U., Brunekreef, B., 2016. Classroom ventilation and indoor air quality-results from the FRESH intervention study. Indoor Air 26, 538–545. https://doi.org/10.1111/ina.12231

Rosbach, J.T.M., Vonk, M., Duijm, F., van Ginkel, J.T., Gehring, U., Brunekreef, B., 2013. A ventilation intervention study in classrooms to improve indoor air quality: the FRESH study. Environ. Health 12, 110. https://doi.org/10.1186/1476-069X-12-110

Smedje, G., Mattsson, M., Wålinder, R., 2011. Comparing mixing and displacement ventilation in classrooms: Pupils perception and health. Indoor Air 21, 454–461. https://doi.org/10.1111/j.1600-0668.2011.00725.x

Smith, M., Hawley, G., Mackay, L., Hosking, J., Mackie, H., Ikeda, E., Egli, V., Ellaway, A., Witten, K., 2020. Impact of Changing Road Infrastructure on Children's Active Travel: A Multi-Methods Study from Auckland, New Zealand. J. Transp. Heal. 18.

Stewart, O., Moudon, A.V., Claybrooke, C., 2014. Multistate evaluation of safe routes to school programs. Am. J. Health Promot. 28, S89-96. https://doi.org/10.4278/ajhp.130430-QUAN-210

Taylor, A.F., Butts-Wilmsmeyer, C., 2020. Self-regulation gains in kindergarten related to frequency of green schoolyard use. J. Environ. Psychol. 70, 101440. https://doi.org/10.1016/j.jenvp.2020.101440

Trompetter, W.J., Boulic, M., Ancelet, T., Garcia-Ramirez, J.C., Davy, P.K., Wang, Y., Phipps, R., 2018. The effect of ventilation on air particulate matter in school classrooms. J. Build. Eng. 18, 164–171. https://doi.org/10.1016/j.jobe.2018.03.009

Twardella, D., Matzen, W., Lahrz, T., Burghardt, R., Spegel, H., Hendrowarsito, L., Frenzel, A.C., Fromme, H., 2012. Effect of classroom air quality on students' concentration: Results of a cluster-randomized cross-over experimental study. Indoor Air 22, 378–387. https://doi.org/10.1111/j.1600-0668.2012.00774.x

Van Den Berg, A., Warren, J.L., McIntosh, A., Hoelscher, D., Ory, M.G., Jovanovic, C., Lopez, M., Whittlesey, L., Kirk, A., Walton, C., McKyer, L., Ranjit, N., 2020. Impact of a Gardening and Physical Activity Intervention in Title 1 Schools: The

TGEG Study. Child. Obes. 16, S44–S54. https://doi.org/10.1089/chi.2019.0238

van den Berg, A.E., Wesselius, J.E., Maas, J., Tanja-Dijkstra, K., 2017. Green Walls for a Restorative Classroom Environment: A Controlled Evaluation Study. Environ. Behav. 49, 791–813. https://doi.org/10.1177/0013916516667976

van Dijk-Wesselius, J.E., Maas, J., Hovinga, D., van Vugt, M., van den Berg, A.E., 2018. The impact of greening schoolyards on the appreciation, and physical, cognitive and social-emotional well-being of schoolchildren: A prospective intervention study. Landsc. Urban Plan. 180, 15–26. https://doi.org/10.1016/j.landurbplan.2018.08.003

Wells, N.M., Myers, B.M., Henderson, C.R.J., 2014. School gardens and physical activity: a randomized controlled trial of low-income elementary schools. Prev. Med. (Baltim). 69 Suppl 1, S27-33. https://doi.org/10.1016/j.ypmed.2014.10.012

Wood, C., Gladwell, V., Barton, J., 2014. A repeated measures experiment of school playing environment to increase physical activity and enhance self-esteem in UK school children. PLoS One 9. https://doi.org/10.1371/journal.pone.0108701
