# Supplementary material for: School-Based Interventions to Support Healthy Indoor and Outdoor Environments for Children: A Systematic Review
Source: Int J Environ Res Public Health. 2023 Jan 18;20(3):1746. doi: 10.3390/ijerph20031746 (PMC9914556; doi:10.3390/ijerph20031746)
Supplement: Supplementary file 1 [file ijerph-20-01746-s001.zip › Supplementary Material S4 risk of bias and study quality tool for intervention studies.pdf]

Supplementary Material S4: Twohig-Bennett and Jones risk of bias and study quality tool for intervention studies

Table S2: Quality Assessment Criteria

**Item**<br>**Methodological quality**	**Description**	**Scale**
1. Reporting: hypothesis	Is the hypothesis/aim/objective of the study clearly described?	1: Yes – clearly described<br>0: No
2. Reporting: outcome(s)	Are the main outcomes to be measured clearly described in the introduction or methods section? (if the main outcomes are first mentioned in the results section, this question should be answered no)	1: Yes – clearly described in introduction/methods<br>0: No – not clearly described/first mentioned in results
3. Reporting: intervention	Are the interventions of interest (air pollution, noise, greenspace, active travel and control or otherwise) clearly described?	1: Yes – clearly described<br>0: No
4. Randomisation	Was there sufficient description of a randomisation process or statistical test to show that comparability between the two groups has been adjusted for (no explanation scores zero)?	1: Yes – description of a randomisation<br>process<br>0: No – no explanation
5. Exposure	Did the authors show that there was no evidence of a concurrent intervention which could have influenced the results (no explanation scores zero)?	1: Yes<br>0: No – no explanation<br>N: Insufficiently described
6. Representativeness	Were the study samples shown to be representative of the study population?	1: Yes – shown to be representative<br>0: No – shown not to be representative<br>N: Insufficiently described
7. Comparability	Were baseline characteristics of the intervention comparable with the control or were potential confounders at baseline approximately adjusted for in analysis?	1: Yes<br>0: No<br>N: Insufficiently described
8. Attrition	Were numbers of participants at follow-up identifiable as at least 80% of the baseline?	1: Yes<br>0: No<br>N: Insufficiently described
9. Outcome assessment: tools	Were valid and reliable tools used to assess participant outcomes?	1: Yes<br>0: No<br>N: Insufficiently described
10. Follow-up time scale	Was the length of time to follow up assessment appropriate for the intervention?	1: Yes<br>0: No
11. Precision of the results	Were confidence intervals or p-values given?	1: Yes<br>0: No

Table S3: Quality appraisal results for intervention studies using the Twohig-Bennett and Jones (2018) risk of bias and study quality tool

	Adapted risk of bias checklist results										
**Reference**	**1. Reporting: hypothesis**	**2. Reporting: outcome(s)**	**3. Reporting: intervention**	**4. Randomisation**	**5. Exposure**	**6. Representativeness**	**7. Comparability**	**8. Attrition**	**9. Outcome assessment tools**	**10. Follow-up time scale**	**11. Precision of the results**	**Total score (out of 11)**
**Air Pollution interventions (n=10)**												
Oh et al., 2014	1	1	0	0	0	N	0	1	1	1	1	6
Pacitto et al., 2020	1	1	0	0	N	0	0	1	1	1	0	5
MacNeill et al., 2016	1	1	0	0	N	0	1	1	1	1	1	7
Bakó-Biró et al., 2012	1	1	0	0	N	N	1	0	1	0	1	5
Smedje et al., 2011	1	1	0	0	N	0	N	0	1	1	1	5
Trompetter et al., 2018	1	1	0	0	N	N	N	1	1	1	0	5
Rosbach et al., 2013	1	1	0	1	1	N	1	0	1	1	1	8
Rosbach et al., 2016	1	1	0	1	1	N	1	0	1	1	1	8
Twardella et al., 2012	1	1	0	1	N	N	1	1	1	1	1	8
Petersen et al., 2016	1	1	0	0	1	N	1	1	N	1	1	7
**Green Spaces (n=17)**												
Block et al., 2012	1	1	0	1	1	N	1	0	1	0	1	7
Wells et al., 2014	1	1	0	1	N	0	1	0	1	1	1	7
Van Den Berg et al., 2020	1	1	0	1	1	N	1	0	1	1	1	8
Rees-Punia et al., 2017	1	1	0	0	N	N	1	1	1	1	1	7
Gustafsson et al., 2012	1	1	0	1	N	N	1	0	1	0	1	6
Largo-Wight et al., 2018	1	1	0	0	N	0	1	1	1	0	1	6
Kuo et al., 2018	1	1	0	1	N	N	1	1	N	0	1	6
Taylor and Butts-Wilmsmeyer, 2020	1	1	0	0	N	N	1	N	1	0	1	5
Bang et al., 2018	1	1	0	1	N	N	1	1	1	0	1	7

Quibell et al., 2017	1	1	0	1	N	N	1	N	1	0	1	6
Amicone et al., 2018	1	1	0	1	N	N	1	N	1	1	1	7
	**Adapted risk of bias checklist results**											
**Reference**	**Reporting: hypothesis**	**Reporting: outcome(s)**	**Reporting: intervention**	**Randomisation**	**Exposure**	**Representativeness**	**Comparability**	**Attrition**	**Outcome assessment tools**	**Follow-up time scale**	**Precision of the results**	**Total score (out of 11)**
Barton et al., 2015	1	1	0	1	N	N	1	1	1	1	1	8
Wood et al., 2014	1	1	0	1	N	N	0	0	1	1	1	6
Raney et al., 2019	1	1	0	0	0	0	N	0	1	1	1	5
van Dijk-Wesselius et al., 2018	1	1	0	1	N	N	1	0	1	1	1	7
van den Berg et al., 2017	1	1	0	1	N	0	1	1	1	1	1	8
Bernardo et al., 2021 (study 1)	1	1	0	1	0	N	1	N	1	1	1	7
Bernardo et al., 2021 (study 2)	1	1	0	0	0	N	N	N	1	1	1	5
**Active Travel (n=12)**												
McDonald et al., 2013	1	1	0	1	N	N	1	N	N	1	1	6
McDonald et al., 2014	1	1	0	1	N	N	1	N	N	1	1	6
Stewart et al., 2014	1	1	0	0	1	0	0	N	N	0	1	4
Hoelscher et al., 2016	1	1	0	1	1	N	1	N	1	1	1	8
Hinckson and Badland, 2011	1	1	0	0	N	N	1	1	0	1	1	6
Hinckson et al., 2011	1	1	0	0	1	N	1	N	0	1	1	6
Ragland et al., 2014	1	0	0	0	N	N	N	N	N	1	0	2
Buliung et al., 2011	1	1	0	0	0	0	0	0	N	0	0	2
Ostergaard et al., 2015	1	1	0	1	1	N	1	1	N	0	1	7
Smith et al., 2020	1	1	0	0	1	N	0	N	N	0	0	3
Mammen et al., 2014a	1	1	0	0	0	0	0	0	N	0	1	3
Mammen et al., 2014b	1	1	0	0	N	N	0	0	1	0	1	4
